# Core members and differential abundance of chrysomelid microbiota in the life stages of *Podontiaaffinis* (Galerucinae) and adult *Silanafarinosa* (Cassidinae, Coleoptera)

**DOI:** 10.3897/BDJ.10.e87459

**Published:** 2022-10-07

**Authors:** Sze-Looi Song, Hoi-Sen Yong, Kah-Ooi Chua, Praphathip Eamsobhana, Phaik-Eem Lim, Kok-Gan Chan

**Affiliations:** 1 Institute for Advanced Studies, Universiti Malaya, Kuala Lumpur, Malaysia Institute for Advanced Studies, Universiti Malaya Kuala Lumpur Malaysia; 2 Institute of Ocean and Earth Sciences, Universiti Malaya, Kuala Lumpur, Malaysia Institute of Ocean and Earth Sciences, Universiti Malaya Kuala Lumpur Malaysia; 3 Institute of Biological Sciences, Faculty of Science, Universiti Malaya, Kuala Lumpur, Malaysia Institute of Biological Sciences, Faculty of Science, Universiti Malaya Kuala Lumpur Malaysia; 4 Centre for Research in Biotechnology for Agriculture, Universiti Malaya, Kuala Lumpur, Malaysia Centre for Research in Biotechnology for Agriculture, Universiti Malaya Kuala Lumpur Malaysia; 5 Department of Parasitology, Faculty of Medicine Siriraj Hospital, Mahidol University, Bangkok, Thailand Department of Parasitology, Faculty of Medicine Siriraj Hospital, Mahidol University Bangkok Thailand

**Keywords:** Bacterial OTUs, Chrysomelidae, phytophagous, 16S rRNA gene amplicon sequencing

## Abstract

The chrysomelid beetles *Podontiaaffinis* and *Silanafarinosa* are members of the subfamilies Galerucinae and Cassidinae, respectively. This study, based on 16S rRNA gene-targeted metagenomics sequencing, reports the core members and differential abundance of bacterial communities in the larvae and adult beetles of *P.affinis* and the adult *S.farinosa*. Cyanobacteria/Melainabacteria group was the predominant phylum in the larvae of *P.affinis*, while Proteobacteria was the predominant phylum in adult *P.affinis* and *S.farinosa*. The number of Order, Family, Genus and Species OTUs in the adult stage of *P.affinis* was higher than that in the larval stage. The bacterial species richness of adult *P.affinis* was significantly higher than that of adult *S.farinosa*. Betaproteobacteria was the predominant class in adult *P.affinis*, Cyanobacteria in the larvae of *P.affinis* and Gammaproteobacteria in *S.farinosa*. The larvae and adult beetles of *P.affinis* and adult *S.farinosa* had a low number of unique and shared bacterial OTUs (> 5% relative abundance). The differences in the microbiota indicate possible differences in nutrient assimilation, host taxonomy and other stochastic processes. These findings provide new information to our understanding of the bacteria associated with specialist phytophagous chrysomelid beetles and beetles in general.

## Introduction

Beetles of the Chrysomelidae family are represented by over 35,000 described species worldwide (Jolivet and Verma 2009). They are predominantly phytophagous. Some species are important crop pests; some are host-plant specialists, while others are host-plant generalists. As the plant cell wall contains cellulose and pectin, the beetles need to digest them to obtain the nutrients. Many chrysomelid and other herbivorous beetles possess genes for producing plant cell wall degrading enzymes (PCWDEs – cellulases and pectinases) ([Bibr B7888201][Bibr B7888179], Kirsch et al. 2014). In addition, their gut microbiota is also a diverse source of PCWDEs. A notable pectinolytic symbiont is ‘‘*Candidatus *Stammera capleta’’ associated with the leaf beetle (*Cassidarubiginosa*) (Salem et al. 2017).

Although economically important, there appears few studies on the microbiota of chrysomelid beetles. Most of the beetles studied are host-plant generalists, such as members of the Cassidinae subfamily – *Cassidarubiginosa* (Salem et al. 2017), *Cephaloleia*spp. (Blankenchip et al. 2018), *Octodontanipae* (Ali et al. 2018, Ali et al. 2019) and *Dactylispaxanthospila* (Cui et al. 2021). Other subfamilies and their species are: Alticinae* – Aphthonavenustula* (Kolasa et al. 2019); Bruchinae – *Callosobruchusmaculatus* (Akami et al. 2019); Chrysomelinae –*Leptinotarsadecemlineata* (Muratoglu et al. 2011) and* Colaphellus bowringi *(Liu et al. 2016); Criocerinae* – Crioceris duodecimpunctata *and* Crioceris quatuordecimpunctata *(Kolasa et al. 2019); Cryptocephalinae –*Cheilotomamusciformis* (Kolasa et al. 2019), *Cryptocephalus* spp. (Montagna et al. 2015); and Gelerucinae –* Diabrotica virgifera* (Chu et al. 2013). The study of microbes that live inside insects has become progressively important in entomological research. However, no study has been reported on the diversity and differences of the microbiota between specialist phytophagous chrysomelid beetles and bettles in general.

We report here the microbiota associated with the larvae and adult females of the leaf beetle *Podontiaaffinis* (Gröndal 1808), a member of Galerucinae and the adult females of *Silanafarinosa* (Boheman 1856), a member of Cassidinae subfamily. These beetles are host-specialists. The adults and larvae of both species are phytophagous. The host plant of *P.affinis* is the golden apple *Spondiasdulcis* (syn. *Spondiascytherea*) of the Anacardiaceae family (Kalshoven 1981); this plant is native to the Pacific, but has been introduced into tropical areas of both the Old and New World (Mitchell and Daly 2015). The host plants for *S.farinosa* are the curry tree* Murraya koenigii* (Rutaceae) (Sajap and Mohamedsaid 1997) and *Ziziphus* sp. (Rhamnaceae) (Takixawa, 1980).

## Materials and Methods

### Sample Collection and DNA Extraction

The larvae and adult female beetles of *P.affinis* were collected from the host plant the golden apple *S. dulcis,* while the adult female beetles of *S.farinosa* were collected from the curry tree* M. koenigii* in the garden of the Institute of Biological Sciences, Universiti Malaya, Malaysia (3°07ʹ9.00ʺN, 101°39ʹ13.79ʺE). Individual specimens were immediately rinsed and preserved in absolute ethanol and stored in a −20°C deep freezer until used for sequencing. Both species are not endangered or protected by law. No permission is needed to collect and study these specialist beetles which are pests of crop plants.

The specimens were surface sterilised twice with 1% sodium hypochlorite solution for 30 seconds followed by washing three times with ultra-high purity water. DNA extraction was performed on the entire specimen using a G-spin^TM^ Total DNA Extraction Mini Kit (iNtRON Biotechnology, Inc, Korea) according to the manufacturer’s instructions with minor modifications – the incubation time of lysate was prolonged to 1 h to ensure complete lysis of the beetles sample.

### Targeted Metagenomics Sequencing

Amplification and sequencing of the V3–V4 region of 16S rRNA gene were carried out as earlier described (Yong et al. 2017a, Yong et al. 2017b, Yong et al. 2019). In short, Q5 Hot Start High-Fidelity PCR 2× Master Mix was used for the initial polymerase chain reaction (PCR) according to the manufacturer’s two-step cycling standard protocol. The primer pair for amplifying the V3–V4 region of the bacterial 16S rRNA gene sequences was MiSeq341F (5ʹ-TCGTCGGCAGCGTCAGATGTGTATAAGAGACAGCCTACGGGNGGCWGCAG-3ʹ) and MiSeq785R (5ʹ-GTCTCGTGGGCTCGGAGATGTGTATAAGAGACAGGACTACHVGGGTATCTAATCC-3ʹ). The 5ʹ-ends are the Illumina adapter sequences, while the 3ʹ-ends of the primers amplify the V3–V4 region of the 16S gene (Klindworth et al. 2013).

### Bioinformatics and Statistical Analysis

Bioinformatics was performed as described in earlier studies (Yong et al. 2017a, Yong et al. 2017b, Yong et al. 2019). UCHIME (Edgar 2010, Edgar et al. 2011) was used to identify and remove potential chimeric sequences. UCLUST (Qiime v.1.9.0) was used to cluster the sequence reads into Operational Taxonomic Units (OTUs) at 97% similarity (Edgar 2010, Caporaso et al. 2010). A representative sequence for each OTU was selected for taxonomic assignment with reference to the National Center for Biotechnology Information (NCBI) 16S microbial database (Yong et al. 2017a, Yong et al. 2017b, Yong et al. 2019). Alpha and beta diversity analyses were performed on Qiime using the default parameters. One-way ANOVA with post-hoc Tukey HSD test was used to compare the mean relative abundance of OTUs of different samples.

## Results

The number of demultiplexed paired-end reads and quality-filtered reads after chimera removal and removal of OTUs with less than 0.01% of total abundance varied within and across the life stages of *P.affinis* and *S.farinosa* (Suppl. material 1). The quality of filtered reads was not significantly different amongst the beetle samples – *P.affinis* larvae: 70359 ± 4472 (mean ± standard deviation) reads, range 65870–74814; *P.affinis* adult: 74545 ± 5902 reads, range 70371–78718; *S.farinosa* adult female: 80224 ± 7315 reads, range 69980–90131; (ANOVA *F* = 2.29, *p* = 0.17) (Suppl. material 1). The larva beetle sample PAL4 had the lowest number of reads (65870) after quality-filtering, while highest number of reads occurred in adult beetle sample SF2 (90131) after quality-filtering (Suppl. material 1).

Eight bacterial phyla (Acidobacteria, Actinobacteria, Bacteroidetes, Cyanobacteria/Melainabacteria group, Deinococcus-Thermus, Firmicutes, Planctomycetes, Proteobacteria) were detected at 97% similarity and filtering at 0.01% abundance (Suppl. materials 2, 3, 4). The other bacterial OTUs comprised of 14 classes, 28 orders, 35 families, 48 genera and 72 putative species. Table 1 summarises the number of OTUs in the larvae and adult beetles. The number of Order, Family, Genus and Species OTUs in the adult stage was higher than that in the larval stage (Table 1).

**Figure 1.**
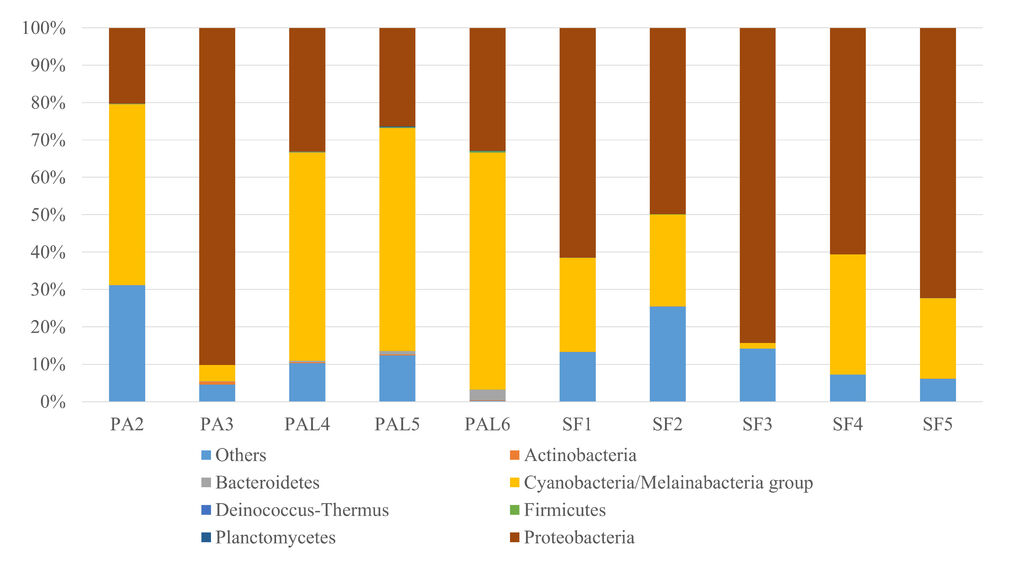
Relative abundance (%) of bacterial phyla in samples of the larvae and adult beetles of *Podontiaaffinis* and adult *Salina farinosa*. PAL4–PAL6, *P.affinis* larva; PA2–PA3, *P.affinis* adult female; SF1–SF5, *S.farinosa* adult female.

Of the eight bacterial phyla, five (Actinobacteria, Bacteroidetes, Cyanobacteria/Melainabacteria group, Planctomycetes, Proteobacteria) were represented in all the beetle specimens, forming the core members of the bacterial community (Table 1, Fig. 1, Suppl. materials 2, 3, 4). Cyanobacteria/Melainabacteria group and Proteobacteria were the dominant phyla, with relative abundance of ≥ 5% in all the specimens, excepting one adult specimen with 4.15% (Suppl. material 2). The other phyla had low relative abundance of less than 1% for all the specimens, excepting one specimen with 2.95% relative abundance for Bacteroidetes (Suppl. material 2).

The larvae had higher mean relative abundance than the adult beetles for Bacteroidetes, Cyanobacteria/Melainabacteria group and Planctomycetes, while the adult beetles had higher mean relative abundance for Actinobacteria and Proteobacteria (Suppl. material 5). Cyanobacteria/Melainabacteria group was the predominant phylum in the larvae, while Proteobacteria was the predominant phylum in the adult beetles.

Five bacterial phyla (Actinobacteria, Bacteroidetes, Cyanobacteria/Melainabacteria group, Firmicutes, Proteobacteria) were detected in the adult beetles (Table 1, Suppl. materials 3, 4). The other OTUs consisted of nine classes, 18 orders, 20 families, 30 genera and 41 putative species.

Cyanobacteria/Melainabacteria group and Proteobacteria were the dominant and core phyla (represented in all specimens with at least one specimen having relative abundance of ≥ 5%). Proteobacteria was the predominant phylum (Suppl. material 6).

The five core bacterial classes were Actinobacteria, Cyanobacteria, Alphaproteobacteria, Betaproteobacteria and Gammaproteobacteria (Suppl. material 6). Cyanobacteria and Gammaproteobacteria were the dominant classes and Gammaproteobacteria was the predominant class.

Of the eight core bacterial orders, Enterobacteriales (56.50 ± 16.75%) was the predominant OTU and Nostocales was the second dominant order (Suppl. material 6). Amongst the 12 core families (Suppl. material 3), Enterobacteriaceae was the predominant OTU and Hapalosiphonaceae was the second dominant order (Suppl. material 6).

Four (*Fischerella*, *Mastigocoleus*, *Kluyvera*, *Pantoea*) out of the 20 core bacterial genera were the dominant OTUs, with *Pantoea* forming the predominant genus (Suppl. materials 3, 6). There were 24 core putative species (Suppl. material 3). The dominant species were *Fischerella* *thermalis*, *Mastigocoleustestarum*, *Kluyveracryocrescens* and *Pantoeaseptica*; *P.septica* was the predominant species with mean relative abundance of 22.09 ± 21.91% (Suppl. material 6).

**Figure 2.**
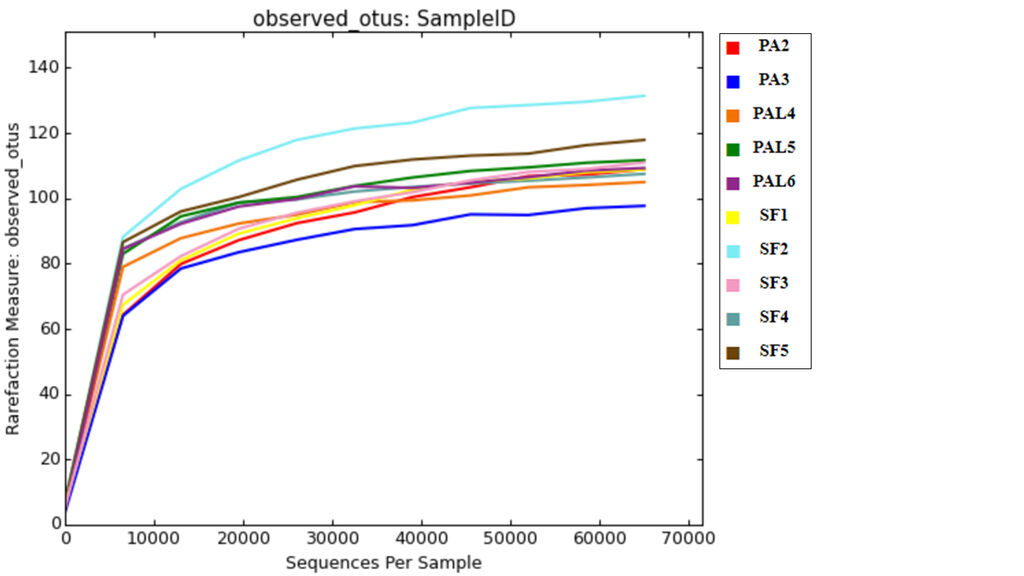
Rarefaction analysis of bacterial communities in the larvae and adult beetles of *Podontiaaffinis* and adult *Salina farinosa*. PAL4–PAL6, *P.affinis* larva; PA2–PA 3, *P.affinis* adult female; SF1–SF5, *S.farinosa* adult female.

**Figure 3.**
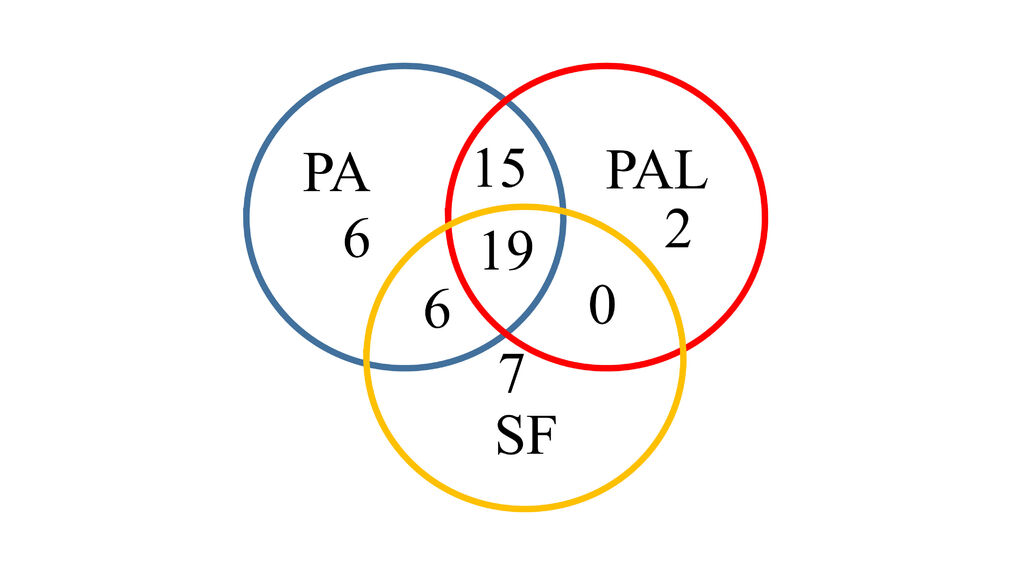
Venn diagrams showing unique and shared bacterial genera in the larvae and adult female beetles of *Podontiaaffinis* and adult female *Salina farinosa*. PAL4–PAL6, *P.affinis* larva; PA2–PA3, *P.affinis* adult female; SF1–SF5, *S.farinosa* adult female.

**Figure 4.**
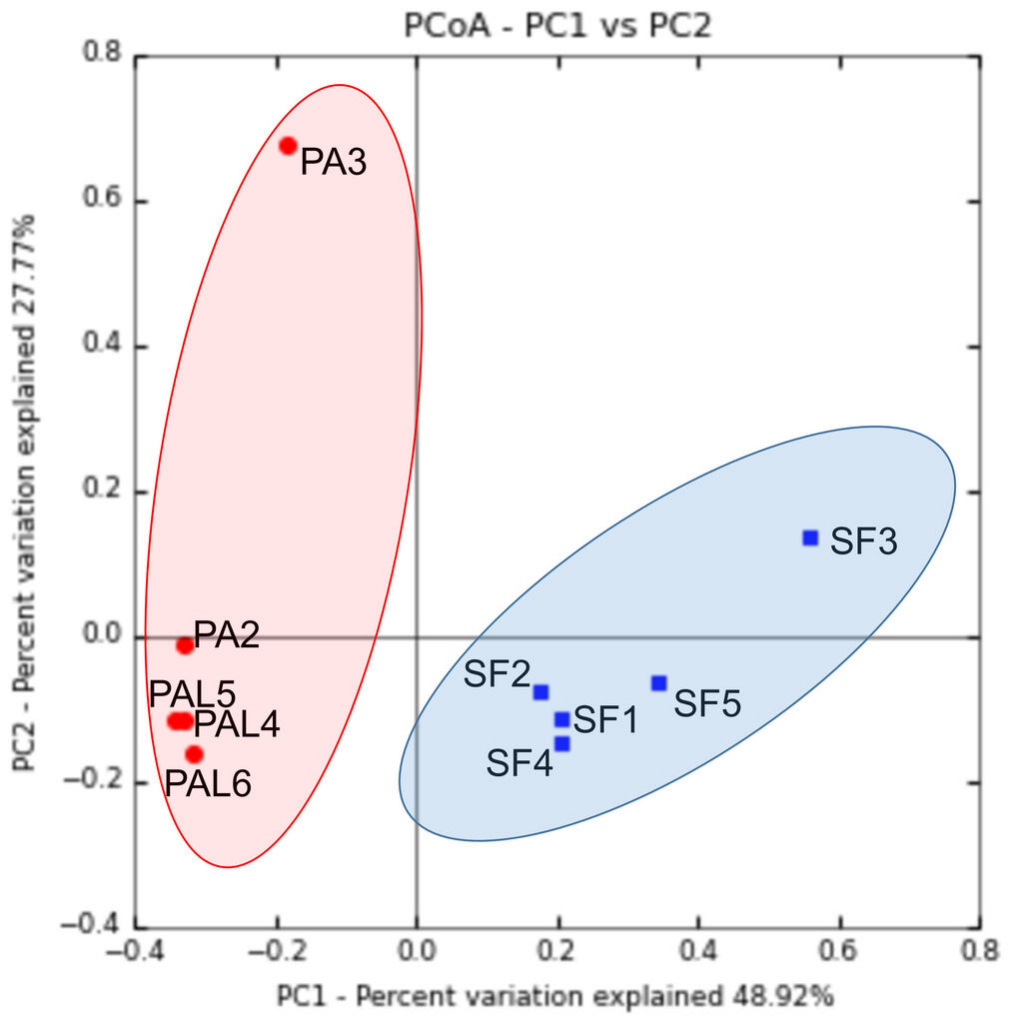
Beta diversity analysis, based on Bray-Curtis dissimilarities of bacterial community associated with the larvae and adult beetles of *Podontiaaffinis* and adult *Salina farinosa*. PAL4–PAL6, *P.affinis* larva; PA2–PA 3, *P.affinis* adult female; SF1–SF5, *S.farinosa* adult female.

Based on rarefaction analysis on a subsample size of 65000 sequences, the number of observed OTUs reached a plateau for all samples at approximately 30000 sequences (Fig. 2). This showed that the 16S rRNA gene amplicon sequencing had obtained adequate coverage in elucidating the bacterial community of *P.affinis* and *S.farinosa* samples in this study. In general, the bacterial OTU diversity varied within and between the larvae and adult beetles of *P.affinis,* as well as the adult *S.farinosa* (Table 2 , Fig. 3). The richness also varied within and between the adult samples of *P.affinis* and *S.farinosa*. The bacterial community in the larvae (mean Shannon index = 3.24 ± 0.24; mean Simpson index = 0.80 ± 0.03) was more diverse than the adult beetles (mean Shannon index = 2.62 ± 0.42; mean Simpson index = 0.75 ± 0.11) of *P.affinis* (Fig. 3, Table 2). Non-parametric statistical test analysis of similarity (ANOSIM) showed significant differences in bacterial diversity between the larva and adult *P.affinis* as well as adult *S.farinosa* (p = 0.01; R = 0.74; number of permutations = 999). The mean bacterial diversity in the *P.affinis* larvae was not significantly different from the adult *P.affinis* and *S.farinosa* beetles – ANOVA with Tukey HSD test: Shannon index Q = 2.98, p = 0.16 and Q = 1.88, p = 0.43, respectively. However the mean diversity was significantly different between *P.affinis* and *S.farinosa* adults – Q = 4.90, p = 0.02. Although the adult sample PA3 exhibited greater variation from other adult and larvae samples, the remaining *P.affinis* samples were highly similar in bacterial community as indicated by close clustering of the samples (PA2, PAL4-PAL6) in beta-diversity analysis, based on Bray-Curtis dissimilarity (Fig. 4). More importantly, all the *P.affinis* and *S.farinosa* samples were clearly differentiated, based on their bacterial community as shown in PCoA of beta-diversity plotted using the Bray-Curtis dissimilarity matrix (Fig. 4).

## Discussion

An earlier study of 62 insect species, including four beetle species, indicated low bacterial species richness and little conclusive evidence that insect taxa other than termites and hymenopterans maintained distinct microbial communities (Colman et al. 2012). In the present study, whether the significantly higher bacterial species richness of adult *P.affinis* than that of adult *S.farinosa* was due to host plants need to be studied. The species richness of the adults and larvae of *P.affinis* was not significantly different. In *O.nipae,* the bacterial diversity varied significantly across the life stages (Ali et al. 2019). The diversity of the core microbiota of six closely-related Cassidinae species of Costa Rican *Cephaloleia* beetles was significantly higher in specialist species, compared to generalists (Blankenchip et al. 2018).

In a recent study of 24 beetle species representing five families (Carabidae, Chrysomelidae, Curculionidae, Scarabaeidae, Staphylinidae) belonging to three trophic guilds (carnivorous, herbivorous, detrivorous), the bacterial communities varied greatly in beetle hosts and the bacterial diversity was shaped by both host phylogenetic relationships (host taxonomy) and trophic affinity (Kolasa et al. 2019). The OTU diversity of Cassidinae (*S.farinosa*) and Galerucinae (*P.affinis*) in the present study is lower than that of the chrysomelid subfamily Alticinae (*Aphthonavenustula*) (Kolasa et al. 2019) and Bruchinae (*Callosobruchusmaculatus*) (Akami et al. 2019), but higher than that of the chrysomelid subfamily Cryptocephalinae (*Cheilotomamusciformis*) (Kolasa et al. 2019)*. * Higher bacterial diversity had been reported for the Cryptocephalinae genus *Cryptocephalus* (Montagna et al. 2015).

Amongst the microbiota of chrysomelid beetles, Proteobacteria, Tenericutes and Firmicutes were the dominant components in Cryptocephalinae genus *Cryptocephalus*(*C.* *acquitanus*, *C.marginellus* and *C.zoiai*) (Montagna et al. 2015). The most dominant bacterial phyla in *Callosobruchus* *maculatus* (Bruchinae) were Proteobacteria, Bacteroidetes and Firmicutes (Akami et al. 2019). Proteobacteria, Actinobacteria and Firmicutes were the dominant bacterial phyla in *Octodontanipae* (Cassidinae) (Ali et al. 2019). It is evident that the bacterial communities at the phylum level vary between, as well as within, the chrysomelid beetle hosts at the species and subfamily level.

The differential predominant bacterial phylum in *P.affinis* larvae and adult beetles in the present study differs from the two major phyla (Proteobacteria and Actinobacteria) present in all developmental stages of *O.nipae* (Ali et al. 2019), indicating a difference between members of Galerucinae and Cassidinae.

The low number of unique and shared bacterial OTUs (> 5% relative abundance) between the larvae and adult beetles of *P.affinis* and between *P.affinis* and *S.farinosa* (Table 3) is similar to the limited core microbiota of six closely-related species of Costa Rican *Cephaloleia* beetles (Chrysomelidae, Cassidinae) (Blankenchip et al. 2018).

In the present study, the microbiome of *S.farinosa* had a high relative abundance of unclassified Enterobacteriaceae (25.25 ± 17.68%, range: 7.70–51.03%). A high proportion of unidentified Enterobacteriaceae (12% of the total recovered sequences) was also recorded in the Cassidinae *Cephaloleia* beetles found on native plants (Blankenchip et al. 2018). Enterobacteriaceae was the most abundant family during the early developmental stages of *O.nipae*, while Anaplasmataceae dominated the adult stage (Ali et al. 2019). Moraxellaceae, Enterobacteriaceae and Pseudomonadaceae were highly prevalent in specialist species of *Cephaloleia* beetles (Blankenchip et al. 2018). Of these bacterial families, Enterobacteriaceae and Pseudomonadaceae were recovered in *S.farinosa*.

The symbiotic bacteria *Pseudomonas*, *Enterobacter* and *Pantoea* (also Enterobacteriaceae) have been found to play influential roles in development, nutrition and success in herbivorous beetles (Blankenchip et al. 2018). *Leptinotarsajuncta* (Chrysomelinae) use *Enterobacter* sp. and *Pantoea* sp. to suppress host plant defences (Wang et al. 2016). *Pantoea* was the most abundant genus in the larval and pupal stages of *O.nipae* (Ali et al. 2019). In the present study, *Pantoea* is a dominant genus in *S.farinosa*. *Enterobacter* and *Pseudomonas* occur at low relative abundance. Studies are needed to determine the widespread use of symbiotic bacteria by herbivorous beetles (and other insects) to cope with host plant defences.

There are core members and differential abundance of the bacterial communities in the life stages of the chrysomelid beetle *P.affinis,* as well as between *P.affinis* (Galerucinae) and *S.farinosa* (Cassidinae). The bacterial species richness of adult *P.affinis* is significantly higher than that of adult *S.farinosa*. Whether the significant difference is due to host plants need to be studied. Compared to the other studies on microbiota of chrysomelid beetles, the bacterial species richness of *P.affinis* and *S.farinosa* (specialist species) are relatively higher than the generalists. The species richness of the adults and larvae of *P.affinis* is, however, not significantly different. Cyanobacteria/Melainabacteria group is the predominant phylum in the larvae of *P.affinis*, while Proteobacteria is the predominant phylum in adult *P.affinis* and *S.farinosa*. The dominant bacterial phyla in these specialist species are also different from those of generalists. However, both generalist and specialist species have high proportions of unclassified Enterobacteriaceae. Betaproteobacteria is the predominant class in adult *P.affinis*, Cyanobacteria in the larvae of *P.affinis* and Gammaproteobacteia in *S.farinosa*. The differences in the microbiotas indicate possible differences in nutrient assimilation and other stochastic processes.

## Supplementary Material

52DC984E-972B-5F08-9A3E-9E7EA4BEE2F7 10.3897/BDJ.10.e87459.suppl1 Supplementary material 1 Sequence reads of 16S rRNA bacteria Data type Table Brief description Sequence reads of 16S rRNA bacteria associated with the life stages of *Podontiaaffinis* and adult *S.farinosa.* Quality filtered reads include chimera removal. File: oo_689383.docx https://binary.pensoft.net/file/689383 Sze-Looi Song, Hoi-Sen Yong, Kah-Ooi Chua, Praphathip Eamsobhana, Phaik-Eem Lim, Kok-Gan Chan

7EA44C7B-5331-5C08-8D82-2C6B68EE8E29 10.3897/BDJ.10.e87459.suppl2 Supplementary material 2 Relative abundance (%) of the bacterial OTUs, determined by 16S rRNA gene sequencing, in the larvae and female adult beetles of *Podontiaaffinis* Data type Table Brief description Relative abundance (%) of the bacterial OTUs, determined by 16S rRNA gene sequencing, in the larvae and female adult beetles of *Podontiaaffinis* after quality filtering at 0.01% and chimera removal. File: oo_689382.docx https://binary.pensoft.net/file/689382 Sze-Looi Song, Hoi-Sen Yong, Kah-Ooi Chua, Praphathip Eamsobhana, Phaik-Eem Lim, Kok-Gan Chan

992757E9-0A72-50C3-A69A-68A39DC98986 10.3897/BDJ.10.e87459.suppl3 Supplementary material 3 Relative abundance (%) of the bacterial OTUs, determined by 16S rRNA gene sequencing, in the female adult beetles of *Salina farinosa* Data type Table Brief description Relative abundance (%) of the bacterial OTUs, determined by 16S rRNA gene sequencing, in the female adult beetles of *Salina farinosa* after quality filtering at 0.01% and chimera removal. File: oo_689381.docx https://binary.pensoft.net/file/689381 Sze-Looi Song, Hoi-Sen Yong, Kah-Ooi Chua, Praphathip Eamsobhana, Phaik-Eem Lim, Kok-Gan Chan

E0B4EC71-C910-5DF4-9519-15D785E6A4C6 10.3897/BDJ.10.e87459.suppl4 Supplementary material 4 Bacterial OTUs detected in *Podontiaaffinis* female adults (PA), *P.affinis* larvae (PAL) and *Salina farinosa* (SF) female adults Data type Table File: oo_689384.docx https://binary.pensoft.net/file/689384 Sze-Looi Song, Hoi-Sen Yong, Kah-Ooi Chua, Praphathip Eamsobhana, Phaik-Eem Lim, Kok-Gan Chan

E3ED6F1A-72B8-5144-987C-0D8207645B02 10.3897/BDJ.10.e87459.suppl5 Supplementary material 5 Comparisons of relative abundance of bacterial OTUs between the larvae and adult beetles of *Podontiaaffinis* Data type Table File: oo_689385.docx https://binary.pensoft.net/file/689385 Sze-Looi Song, Hoi-Sen Yong, Kah-Ooi Chua, Praphathip Eamsobhana, Phaik-Eem Lim, Kok-Gan Chan

F8AD49B0-960A-5F51-B646-DE65976A1C40 10.3897/BDJ.10.e87459.suppl6 Supplementary material 6 Comparisons of relative abundance of bacterial OTUs between the adult beetles of *Podontiaaffinis* and *Salina farinosa* Data type Table File: oo_689386.docx https://binary.pensoft.net/file/689386 Sze-Looi Song, Hoi-Sen Yong, Kah-Ooi Chua, Praphathip Eamsobhana, Phaik-Eem Lim, Kok-Gan Chan

## Figures and Tables

**Table 1.** Number of bacterial OTUs detected in the larvae and adult female beetles of *Podontiaaffinis* and adult female *Salina farinosa*. +, unclassified OTUs.

OTU	*P.affinis* larva	*P.affinis* adult	*P.affinis* total	*S.farinosa* adult
Phylum	8 (100%)	8 (100%)	8	5
Class	13 (92.86%)	13 (92.86%)	14	9
Order	24 (85.71%)	27 (96.43%)	28	18
Family	32+ (91.43%)	34+ (97.14%)	35+	20+
Genus	36+ (76.60%)	45+ (95.74%)	47+	30+
Species	56 (77.78%)	68+ (94.44%)	72+	41+

**Table 2.** Diversity of bacterial OTUs in the larvae and adult female beetles of *Podontiaaffinis* and adult female *Salina farinosa* revealed by the NGS 16S rRNA gene. PAL4–PAL6, *P.affinis* larva; PA2–PA3, *P.affinis* adult female; SF1–SF5, *S.farinosa* adult female; PD, phylogenetic diversity.

Sample	Shannon	Simpson	PD whole tree	Chao1	Observed OTUs	Goods coverage
PA2	2.91	0.83	8.66	132.13	113	1.00
PA3	2.32	0.67	7.65	99.15	98	1.00
PAL4	3.51	0.83	8.35	110.14	105	1.00
PAL5	3.13	0.80	8.23	120.86	113	1.00
PAL6	3.08	0.78	8.73	122.00	111	1.00
SF1	3.15	0.83	8.01	111.59	111	1.00
SF2	3.61	0.89	9.10	141.11	135	1.00
SF3	3.27	0.84	7.90	116.93	113	1.00
SF4	3.87	0.90	7.52	113.60	108	1.00
SF5	3.88	0.90	8.51	125.63	120	1.00

**Table 3.** Bacterial OTUs with ≥ 5% mean relative abundance in the larvae and adult females of *Podontiaaffinis* and *Salina farinosa* adult females, revealed by the 16S rRNA gene. Values within brackets indicate mean and standard deviation. PA, *P.affinis* adult female; PAL, *P.affinis* larva; SF, *S.farinosa* adult female.

Life stage	Phylum	Class	Order	Family	Genus	Species
PA	Cyanobacteria/ Melainabacteria Group (24.63 ± 28.96)	Cyanobacteria (24.57 ± 29.06)	Nostocales (24.45 ± 29.05)	Hapalosiphonaceae (24.56 ± 29.05)	*Fischerella* (13.43 ± 15.77)	*F.thermalis* (13.43±15.77)
					*Mastigocoleus* (11.13 ± 13.28)	*M.testarum* (11.13±13.28)
	Proteobacteria (50.99 ± 46.08)	Alphaproteobacteria (5.29 ± 3.54)				
		Betaproteobacteria (45.34 ± 42.55)	Burkholderiales (44.44 ± 43.63)	Burkholderiaceae (44.42 ± 43.62)	*Burkholderia* (44.28 ± 43.43)	*B.lata* (44.28±43.43)
PAL	Cyanobacteria/ Melainabacteria Group (58.20 ± 3.85)	Cyanobacteria (58.20 ± 3.85)	Nostocales (58.19 ± 3.85)	Hapalosiphonaceae (58.19 ± 3.85)	*Fischerella* (31.67 ± 2.26)	*F.thermalis* (31.67±2.26)
					*Mastigocoleus* (26.51 ± 1.60)	*M.testarum* (26.51±1.60)
			Rhodospirillales (7.79 ± 2.37)	Rhodospirillaceae (7.74 ± 2.39)	*Limimonas* (7.74 ± 2.39)	*L.halophila* (7.74±2.39)
	Proteobacteria (29.24 ± 3.92)	Alphaproteobacteria (16.23 ± 3.99)				
		Betaproteobacteria (12.58 ± 1.32)	Burkholderiales (6.27 ± 0.50)	Burkholderiaceae (5.52 ± 0.49)	*Burkholderia* (5.51 ± 0.51)	*B.lata* (5.51±0.51)
			Neisseriales (6.31 ± 1.82)	Chromobacteriaceae (6.31 ± 1.82)	*Jeongeupia* (6.31 ± 1.82)	*J.chitinilytica* (6.31±1.82)
SF	Cyanobacteria/ Melainabacteria Group (21.24 ± 11.75)	Cyanobacteria (21.24 ± 11.75)	Nostocales (21.24 ± 11.75)	Hapalosiphonaceae (21.24 ±11.75)	*Fischerella* (11.63 ± 6.41)	*F.thermalis* (11.61±6.41)
					*Mastigocoleus* (9.61 ± 5.34)	*M.testarum* (9.61±5.34)
	Proteobacteria (65.23 ± 12.72)	Gammaproteobacteria (57.03 ± 17.23)	Enterobacteriales (56.50 ± 16.75)	Enterobacteriaceae (56.50 ± 16.75)	*Kluyvera* (7.96 ± 10.79)	*K.cryocrescens* (7.96±10.79)
					*Pantoea* (22.41 ± 22.09)	*P.septica* (22.09±21.91)
