# Supplementary material for: Core members and differential abundance of chrysomelid microbiota in the life stages of Podontiaaffinis (Galerucinae) and adult Silanafarinosa (Cassidinae, Coleoptera)
Source: Biodivers Data J. 2022 Oct 7;10:e87459. doi: 10.3897/BDJ.10.e87459 (PMC9836631; doi:10.3897/BDJ.10.e87459)
Supplement: Supplementary material 1 — Sequence reads of 16S rRNA bacteria [file bdj-10-e87459-s001.docx]

**Table S1**

Sequence reads of 16S rRNA bacteria associated with the life stages of *Podontia affinis* and adult *S. farinosa.* Quality filtered reads include chimera removal. PAL4–PAL6, *P. affinis* larva; PA2–PA4, *P. affinis* adult female; SF1–SF5, *S. farinosa* adult female.

| Sample | Paired-end reads | Quality-filtered reads |
| --- | --- | --- |
| PA2 | 223990 | 78718 |
| PA3 | 212774 | 70371 |
| PAL4 | 189718 | 65870 |
| PAL5 | 210894 | 70393 |
| PAL6 | 226608 | 74814 |
| SF1 | 209054 | 81755 |
| SF2 | 227322 | 90131 |
| SF3 | 195044 | 77643 |
| SF4 | 190416 | 69980 |
| SF5 | 211856 | 81613 |
