# Supplementary material for: Core members and differential abundance of chrysomelid microbiota in the life stages of Podontiaaffinis (Galerucinae) and adult Silanafarinosa (Cassidinae, Coleoptera)
Source: Biodivers Data J. 2022 Oct 7;10:e87459. doi: 10.3897/BDJ.10.e87459 (PMC9836631; doi:10.3897/BDJ.10.e87459)
Supplement: Supplementary material 2 — Relative abundance (%) of the bacterial OTUs, determined by 16S rRNA gene sequencing, in the larvae and female adult beetles of Podontiaaffinis [file bdj-10-e87459-s002.docx]

**Table S2**

Relative abundance (%) of the bacterial OTUs, determined by 16S rRNA gene sequencing, in the larvae and female adult beetles of *Podontia affinis* after quality filtering at 0.01% and chimera removal. PAL4–PAL6, larva; PA2–PA3, adult.

|  | PA2 | PA3 | PAL4 | PAL5 | PAL6 |
| --- | --- | --- | --- | --- | --- |
| **Phylum Acidobacteria** | 0.02 | 0.09 | 0 | 0 | 0.01 |
| **Class Acidobacteriia** | 0.02 | 0.09 | 0 | 0 | 0.01 |
| Order Acidobacteriales | 0.02 | 0.09 | 0 | 0 | 0.01 |
| Family Acidobacteriaceae | 0.02 | 0.09 | 0 | 0 | 0.01 |
| *Terriglobus* | 0.02 | 0.09 | 0 | 0 | 0.01 |
| *Terriglobus tenax* | 0.02 | 0.09 | 0 | 0 | 0.01 |
| **Phylum Actinobacteria** | 0.10 | 0.92 | 0.36 | 0.37 | 0.26 |
| **Class Actinobacteria** | 0.10 | 0.92 | 0.36 | 0.37 | 0.26 |
| Order Frankiales | 0.00 | 0.05 | 0 | 0 | 0 |
| Family Frankiaceae | 0.00 | 0.05 | 0 | 0 | 0 |
| *Jatrophihabitans* | 0.00 | 0.05 | 0 | 0 | 0 |
| *Jatrophihabitans endophyticus* | 0.00 | 0.05 | 0 | 0 | 0 |
| Order Kineosporiales | 0.00 | 0 | 0.05 | 0.07 | 0.03 |
| Family Kineosporiaceae | 0.00 | 0 | 0.05 | 0.07 | 0.03 |
| *Kineococcus* | 0.00 | 0 | 0.05 | 0.07 | 0.03 |
| *Kineococcus endophyticus* | 0.00 | 0 | 0.05 | 0.07 | 0.03 |
| Order Micrococcales | 0.05 | 0.46 | 0.31 | 0.25 | 0.22 |
| Family Microbacteriaceae | 0.02 | 0.44 | 0.31 | 0.24 | 0.21 |
| *Curtobacterium* | 0.02 | 0.44 | 0.31 | 0.24 | 0.21 |
| *Curtobacterium oceanosedimentum* | 0.02 | 0.44 | 0.31 | 0.24 | 0.21 |
| Family Micrococcaceae | 0.04 | 0.03 | 0.00 | 0.01 | 0.00 |
| *Kocuria* | 0.04 | 0.03 | 0.00 | 0.01 | 0.00 |
| *Kocuria palustris* | 0.04 | 0.03 | 0.00 | 0.01 | 0.00 |
| Order Micromonosporales | 0.00 | 0 | 0 | 0.04 | 0.01 |
| Family Micromonosporaceae | 0.00 | 0 | 0 | 0.04 | 0.01 |
| *Catellatospora* | 0.00 | 0 | 0 | 0.04 | 0.01 |
| *Catellatospora coxensis* | 0.00 | 0 | 0 | 0.04 | 0.01 |
| Order Propionibacteriales | 0.01 | 0.06 | 0.00 | 0 | 0 |
| Family Propionibacteriaceae | 0.01 | 0.06 | 0.00 | 0 | 0 |
| *Propionibacterium* | 0.01 | 0.06 | 0.00 | 0 | 0 |
| *Propionibacterium acnes*\|*Propionibacterium acnes* KPA171202 | 0.01 | 0.06 | 0.00 | 0 | 0 |
| Order Pseudonocardiales | 0.02 | 0.35 | 0 | 0 | 0.00 |
| Family Pseudonocardiaceae | 0.02 | 0.35 | 0 | 0 | 0.00 |
| *Actinomycetospora* | 0.00 | 0.25 | 0 | 0 | 0.00 |
| *Actinomycetospora atypica* | 0 | 0.11 | 0 | 0 | 0 |
| *Actinomycetospora chiangmaiensis* | 0.00 | 0.13 | 0 | 0 | 0.00 |
| *Pseudonocardia* | 0.02 | 0.11 | 0 | 0 | 0 |
| *Pseudonocardia ammonioxydans* | 0.01 | 0.06 | 0 | 0 | 0 |
| *Pseudonocardia kujensis* | 0.01 | 0.05 | 0 | 0 | 0 |
| **Phylum Bacteroidetes** | 0.15 | 0.14 | 0.39 | 0.87 | 2.95 |
| **Class Cytophagia** | 0.02 | 0.03 | 0.19 | 0.85 | 2.72 |
| Order Cytophagales | 0.02 | 0.03 | 0.19 | 0.85 | 2.72 |
| Family Cytophagaceae | 0.02 | 0.03 | 0.19 | 0.85 | 2.72 |
| *Hymenobacter* | 0.02 | 0.03 | 0.19 | 0.68 | 1.77 |
| *Hymenobacter aerophilus* | 0 | 0 | 0 | 0.01 | 0.77 |
| *Hymenobacter flocculans* | 0.01 | 0 | 0 | 0.01 | 0.64 |
| *Hymenobacter metalli* | 0.01 | 0.03 | 0.18 | 0.65 | 0.27 |
| *Hymenobacter ocellatus* | 0.01 | 0 | 0.00 | 0.01 | 0.09 |
| *Spirosoma* | 0 | 0 | 0.01 | 0.17 | 0.95 |
| *Spirosoma oryzae* | 0 | 0 | 0.01 | 0.17 | 0.95 |
| **Class Flavobacteriia** | 0 | 0 | 0 | 0 | 0.05 |
| Order Flavobacteriales | 0 | 0 | 0 | 0 | 0.05 |
| Family Flavobacteriaceae | 0 | 0 | 0 | 0 | 0.05 |
| *Chryseobacterium* | 0 | 0 | 0 | 0 | 0.05 |
| *Chryseobacterium gambrini* | 0 | 0 | 0 | 0 | 0.05 |
| **Class Sphingobacteriia** | 0.12 | 0.10 | 0.20 | 0.02 | 0.17 |
| Order Sphingobacteriales | 0.12 | 0.10 | 0.20 | 0.02 | 0.17 |
| Family Sphingobacteriaceae | 0.12 | 0.10 | 0.20 | 0.02 | 0.17 |
| *Mucilaginibacter* | 0.02 | 0.10 | 0.20 | 0.02 | 0.17 |
| *Mucilaginibacter daejeonensis* | 0.00 | 0 | 0.11 | 0.00 | 0.01 |
| *Mucilaginibacter koreensis* | 0.00 | 0.10 | 0.01 | 0.01 | 0.04 |
| *Mucilaginibacter lutimaris* | 0.01 | 0 | 0.08 | 0.01 | 0.13 |
| *Nubsella* | 0.10 | 0 | 0 | 0 | 0 |
| *Nubsella zeaxanthinifaciens* | 0.10 | 0 | 0 | 0 | 0 |
| **Phylum Cyanobacteria/Melainabacteria group** | 45.11 | 4.15 | 54.95 | 57.20 | 62.46 |
| **Class Cyanobacteria** | 45.11 | 4.02 | 54.95 | 57.20 | 62.46 |
| Order Pleurocapsales | 0.00 | 0 | 0.01 | 0.02 | 0.02 |
| Family Chroococcidiopsidaceae | 0.00 | 0 | 0.01 | 0.02 | 0.02 |
| *Chroococcidiopsis* | 0.00 | 0 | 0.01 | 0.02 | 0.02 |
| *Chroococcidiopsis* *thermalis*\|*Chroococcidiopsis thermalis* PCC 7203 | 0.00 | 0 | 0.01 | 0.02 | 0.02 |
| Order Nostocales | 45.10 | 4.02 | 54.94 | 57.18 | 62.44 |
| Family Hapalosiphonaceae | 45.10 | 4.02 | 54.94 | 57.18 | 62.44 |
| *Fischerella* | 24.58 | 2.28 | 29.65 | 31.25 | 34.12 |
| *Fischerella thermalis* | 24.58 | 2.28 | 29.65 | 31.25 | 34.12 |
| *Mastigocoleus* | 20.52 | 1.74 | 25.29 | 25.93 | 28.32 |
| *Mastigocoleus testarum* | 20.52 | 1.74 | 25.29 | 25.93 | 28.32 |
| **Class Cyanobacteria\|Oscillatoriophycideae** | 0 | 0.13 | 0 | 0 | 0 |
| Order Oscillatoriales | 0 | 0.13 | 0 | 0 | 0 |
| Family Pseudanabaenaceae | 0 | 0.13 | 0 | 0 | 0 |
| *Aerosakkonema* | 0 | 0.05 | 0 | 0 | 0 |
| *Aerosakkonema funiforme* | 0 | 0.05 | 0 | 0 | 0 |
| *Tapinothrix* | 0 | 0.07 | 0 | 0 | 0 |
| *Tapinothrix clintonii*\|*Tapinothrix clintonii* GSE-PSE06-07G | 0 | 0.07 | 0 | 0 | 0 |
| **Phylum Deinococcus-Thermus** | 0 | 0.02 | 0 | 0 | 0.03 |
| **Class Deinococci** | 0 | 0.02 | 0 | 0 | 0.03 |
| Order Deinococcales | 0 | 0.02 | 0 | 0 | 0.03 |
| Family Deinococcaceae | 0 | 0.02 | 0 | 0 | 0.03 |
| *Deinococcus* | 0 | 0.02 | 0 | 0 | 0.03 |
| *Deinococcus xinjiangensis* | 0 | 0.02 | 0 | 0 | 0.03 |
| **Phylum Firmicutes** | 0.04 | 0 | 0.12 | 0.16 | 0.29 |
| **Class Clostridia** | 0.04 | 0 | 0.12 | 0.16 | 0.29 |
| Order Clostridiales | 0.04 | 0 | 0.12 | 0.16 | 0.29 |
| Family Heliobacteriaceae | 0.04 | 0 | 0.12 | 0.16 | 0.29 |
| *Heliorestis* | 0.04 | 0 | 0.12 | 0.16 | 0.29 |
| *Heliorestis acidaminivorans* | 0.04 | 0 | 0.12 | 0.16 | 0.29 |
| **Phylum Planctomycetes** | 0.00 | 0.01 | 0.05 | 0.21 | 0.14 |
| **Class Planctomycetia** | 0.00 | 0.01 | 0.05 | 0.21 | 0.14 |
| Order Planctomycetales | 0.00 | 0.01 | 0.05 | 0.21 | 0.14 |
| Family Isosphaeraceae | 0.00 | 0.01 | 0.05 | 0.21 | 0.14 |
| *Aquisphaera* | 0.00 | 0.01 | 0.05 | 0.21 | 0.14 |
| *Aquisphaera giovannonii* | 0.00 | 0.01 | 0.05 | 0.21 | 0.14 |
| **Phylum Proteobacteria** | 18.41 | 83.57 | 31.75 | 24.72 | 31.25 |
| **Class Alphaproteobacteria** | 2.78 | 7.79 | 20.02 | 12.06 | 16.61 |
| Order Caulobacterales | 0.01 | 0.13 | 0.00 | 0.00 | 0 |
| Family Caulobacteraceae | 0.01 | 0.13 | 0.00 | 0.00 | 0 |
| *Phenylobacterium* | 0.01 | 0.13 | 0.00 | 0.00 | 0 |
| *Phenylobacterium koreense* | 0.01 | 0.13 | 0.00 | 0.00 | 0 |
| Order Rhizobiales | 0.46 | 7.30 | 4.48 | 2.76 | 4.51 |
| Family Aurantimonadaceae | 0.01 | 0 | 1.30 | 0.22 | 0.04 |
| *Aureimonas* | 0.01 | 0 | 1.30 | 0.22 | 0.04 |
| *Aureimonas ureilytica*\|*Aureimonas ureilytica* DSM 18598 = NBRC 106430 | 0.01 | 0 | 1.30 | 0.22 | 0.04 |
| Family Bradyrhizobiaceae | 0.12 | 0.24 | 0.01 | 0.11 | 0.49 |
| *Bradyrhizobium* | 0.10 | 0.14 | 0 | 0.00 | 0.01 |
| *Bradyrhizobium ottawaense* | 0.10 | 0.14 | 0 | 0.00 | 0.01 |
| *Salinarimonas* | 0.02 | 0.10 | 0.01 | 0.11 | 0.47 |
| *Salinarimonas rosea* | 0.02 | 0.10 | 0.01 | 0.11 | 0.47 |
| Family Brucellaceae | 0.02 | 0 | 0.00 | 0.10 | 0.03 |
| *Mycoplana* | 0.02 | 0 | 0.00 | 0.10 | 0.03 |
| *Mycoplana ramosa* | 0.02 | 0 | 0.00 | 0.10 | 0.03 |
| Family Methylobacteriaceae | 0.26 | 7.06 | 2.85 | 2.25 | 3.93 |
| *Methylobacterium* | 0.26 | 7.06 | 2.85 | 2.25 | 3.93 |
| *Methylobacterium aerolatum* | 0.00 | 0.03 | 0.07 | 0.05 | 0.02 |
| *Methylobacterium extorquens* group\|*Methylobacterium extorquens*\|*Methylobacterium extorquens* AM1 | 0.03 | 0.00 | 0 | 0.06 | 0.12 |
| *Methylobacterium iners* | 0.00 | 0.05 | 0 | 0 | 0 |
| *Methylobacterium komagatae*\|*Methylobacterium komagatae* DSM 19563 | 0.05 | 0.05 | 0.01 | 0.03 | 0.73 |
| *Methylobacterium phyllostachyos* | 0.12 | 6.76 | 2.53 | 1.73 | 2.05 |
| *Methylobacterium radiotolerans*\|*Methylobacterium radiotolerans* JCM 2831 | 0.00 | 0.06 | 0.08 | 0.09 | 0.01 |
| *Methylobacterium tarhaniae* | 0.06 | 0.11 | 0.15 | 0.30 | 1.01 |
| Family Rhizobiaceae | 0.04 | 0 | 0.31 | 0.08 | 0.02 |
| *Agrobacterium* | 0.04 | 0 | 0.31 | 0.08 | 0.02 |
| *Agrobacterium larrymoorei* | 0.04 | 0 | 0.31 | 0.08 | 0.02 |
| Order Rhodobacterales | 0.06 | 0.00 | 0.02 | 0.02 | 0.02 |
| Family Rhodobacteraceae | 0.06 | 0.00 | 0.02 | 0.02 | 0.02 |
| *Paracoccus* | 0.06 | 0 | 0 | 0 | 0 |
| *Paracoccus aminovorans* | 0.06 | 0 | 0 | 0 | 0 |
| *Roseibium* | 0.00 | 0.00 | 0.02 | 0.02 | 0.02 |
| *Roseibium aquae* | 0.00 | 0.00 | 0.02 | 0.02 | 0.02 |
| Order Rhodospirillales | 2.00 | 0.18 | 5.37 | 7.89 | 10.10 |
| Family Acetobacteraceae | 0.01 | 0 | 0.08 | 0.05 | 0.02 |
| *Roseomonas* | 0.01 | 0 | 0.08 | 0.05 | 0.02 |
| *Roseomonas aerilata* | 0.01 | 0 | 0.08 | 0.05 | 0.02 |
| Family Rhodospirillaceae | 2.00 | 0.18 | 5.30 | 7.84 | 10.08 |
| *Limimonas* | 2.00 | 0.18 | 5.30 | 7.84 | 10.08 |
| *Limimonas halophila* | 2.00 | 0.18 | 5.30 | 7.84 | 10.08 |
| Order Sphingomonadales | 0.25 | 0.17 | 10.13 | 1.39 | 1.98 |
| Family Sphingomonadaceae | 0.25 | 0.17 | 10.13 | 1.39 | 1.98 |
| *Sphingomonas* | 0.25 | 0.17 | 10.13 | 1.39 | 1.98 |
| *Sphingomonas canadensis* | 0.01 | 0.01 | 0.23 | 0.04 | 0.08 |
| *Sphingomonas changbaiensis*\|*Sphingomonas changbaiensis* NBRC 104936 | 0.01 | 0.01 | 0.01 | 0.09 | 0.07 |
| *Sphingomonas echinoides* | 0.01 | 0.01 | 0.01 | 0.01 | 0.03 |
| *Sphingomonas endophytica* | 0.04 | 0.02 | 5.37 | 0.70 | 0.51 |
| *Sphingomonas guangdongensis* | 0 | 0 | 0.03 | 0.01 | 0.03 |
| *Sphingomonas kyungheensis* | 0.09 | 0.05 | 1.09 | 0.18 | 0.58 |
| *Sphingomonas paucimobilis* | 0.03 | 0.00 | 2.53 | 0.20 | 0.29 |
| *Sphingomonas roseiflava* | 0.04 | 0.02 | 0.81 | 0.10 | 0.28 |
| *Sphingomonas wittichii*\|*Sphingomonas wittichii* RW1 | 0.02 | 0.05 | 0.02 | 0.03 | 0.07 |
| *Sphingomonas yunnanensis* | 0.00 | 0 | 0.02 | 0.03 | 0.05 |
| **Class Betaproteobacteria** | 15.25 | 75.43 | 11.25 | 12.59 | 13.89 |
| Order Burkholderiales | 13.59 | 75.29 | 6.80 | 6.21 | 5.80 |
| Family Burkholderiaceae | 13.57 | 75.26 | 5.05 | 6.03 | 5.47 |
| *Burkholderia* | 13.57 | 74.99 | 5.02 | 6.03 | 5.47 |
| *Burkholderia cepacia* complex\|*Burkholderia lata* | 13.57 | 74.99 | 5.02 | 6.03 | 5.47 |
| *Caballeronia* | 0.00 | 0.26 | 0.03 | 0.00 | 0 |
| *Burkholderia megalochromosomata* | 0.00 | 0.26 | 0.03 | 0.00 | 0 |
| Family Comamonadaceae | 0.01 | 0.00 | 0.84 | 0.17 | 0.28 |
| *Ramlibacter* | 0.01 | 0.00 | 0.84 | 0.17 | 0.28 |
| *Ramlibacter solisilvae* | 0.01 | 0.00 | 0.84 | 0.17 | 0.28 |
| Family Oxalobacteraceae | 0.01 | 0.03 | 0.91 | 0.01 | 0.06 |
| *Massilia* | 0.01 | 0.03 | 0.91 | 0.01 | 0.06 |
| *Massilia consociata* | 0.01 | 0.03 | 0.91 | 0.01 | 0.06 |
| Order Neisseriales | 1.66 | 0.14 | 4.45 | 6.38 | 8.09 |
| Family Chromobacteriaceae | 1.66 | 0.14 | 4.45 | 6.38 | 8.09 |
| *Jeongeupia* | 1.66 | 0.14 | 4.45 | 6.38 | 8.09 |
| *Jeongeupia chitinilytica* | 1.66 | 0.14 | 4.45 | 6.38 | 8.09 |
| **Class Deltaproteobacteria** | 0.27 | 0 | 0.00 | 0 | 0.26 |
| Order Myxococcales | 0.27 | 0 | 0.00 | 0 | 0.26 |
| Family Cystobacterineae | 0.27 | 0 | 0.00 | 0 | 0.26 |
| *Cystobacter* | 0.27 | 0 | 0.00 | 0 | 0.26 |
| *Cystobacter*\|*Cystobacter velatus* | 0.27 | 0 | 0.00 | 0 | 0.26 |
| **Class Gammaproteobacteria** | 0.11 | 0.35 | 0.48 | 0.07 | 0.48 |
| Order Oceanospirillales | 0.07 | 0.34 | 0.03 | 0.03 | 0.03 |
| Family Halomonadaceae | 0.07 | 0.34 | 0.03 | 0.03 | 0.03 |
| *Halomonas* | 0.07 | 0.34 | 0.03 | 0.03 | 0.03 |
| *Halomonas stevensii*\|*Halomonas stevensii* S18214 | 0.07 | 0.34 | 0.03 | 0.03 | 0.03 |
| Order Pseudomonadales | 0 | 0.00 | 0.45 | 0.04 | 0.46 |
| Family Moraxellaceae | 0 | 0.00 | 0.00 | 0.00 | 0.38 |
| *Moraxella* | 0 | 0.00 | 0.00 | 0.00 | 0.38 |
| *Moraxella osloensis* | 0 | 0.00 | 0.00 | 0.00 | 0.38 |
| Family Pseudomonadaceae | 0 | 0.00 | 0.44 | 0.04 | 0.07 |
| *Pseudomonas* | 0 | 0.00 | 0.44 | 0.04 | 0.07 |
| *Pseudomonas putida* group\|*Pseudomonas oryzihabitans* | 0 | 0.00 | 0.44 | 0.04 | 0.07 |
| Order Xanthomonadales | 0.04 | 0 | 0 | 0 | 0 |
| Family Xanthomonadaceae | 0.04 | 0 | 0 | 0 | 0 |
| *Stenotrophomonas* | 0.04 | 0 | 0 | 0 | 0 |
| *Stenotrophomonas maltophilia* group\|*Stenotrophomonas* *maltophilia*\|*Stenotrophomonas* *maltophilia* R551-3 | 0.04 | 0 | 0 | 0 | 0 |
| No blast hit | 36.18 | 11.10 | 12.38 | 16.48 | 2.62 |
