# Supplementary material for: Core members and differential abundance of chrysomelid microbiota in the life stages of Podontiaaffinis (Galerucinae) and adult Silanafarinosa (Cassidinae, Coleoptera)
Source: Biodivers Data J. 2022 Oct 7;10:e87459. doi: 10.3897/BDJ.10.e87459 (PMC9836631; doi:10.3897/BDJ.10.e87459)
Supplement: Supplementary material 3 — Relative abundance (%) of the bacterial OTUs, determined by 16S rRNA gene sequencing, in the female adult beetles of Salina farinosa [file bdj-10-e87459-s003.docx]

**Table S3**

Relative abundance (%) of the bacterial OTUs, determined by 16S rRNA gene sequencing, in the female adult beetles of *Salina farinosa* after quality filtering at 0.01% and chimera removal. SF, *Salina farinosa*; PA, *Podontia affinis* adult (included for comparison).

|  | PA2 | PA3 | SF1 | SF2 | SF3 | SF4 | SF5 |
| --- | --- | --- | --- | --- | --- | --- | --- |
| **Phylum Actinobacteria** | 0.10 | 0.92 | 0.01 | 0.02 | 0.02 | 0.01 | 0.00 |
| **Class Actinobacteria** | 0.10 | 0.92 | 0.01 | 0.02 | 0.02 | 0.01 | 0.00 |
| Order Micrococcales | 0.05 | 0.46 | 0.01 | 0.02 | 0.01 | 0.01 | 0.00 |
| Family Microbacteriaceae | 0.02 | 0.44 | 0.01 | 0.02 | 0.01 | 0.01 | 0.00 |
| *Curtobacterium* | 0.02 | 0.44 | 0.00 | 0.01 | 0.00 | 0.00 | 0.00 |
| *Curtobacterium oceanosedimentum* | 0.02 | 0.44 | 0.00 | 0.01 | 0.00 | 0.00 | 0.00 |
| *Leifsonia* | 0.00 | 0.24 | 0.00 | 0.01 | 0.01 | 0.00 | 0.00 |
| *Leifsonia shinshuensis* | 0.00 | 0.24 | 0.00 | 0.01 | 0.01 | 0.00 | 0.00 |
| Order Propionibacteriales | 0.01 | 0.06 | 0.00 | 0.00 | 0.00 | 0.00 | 0.00 |
| Family Propionibacteriaceae | 0.01 | 0.06 | 0.00 | 0.00 | 0.00 | 0.00 | 0.00 |
| *Propionibacterium* | 0.01 | 0.06 | 0.00 | 0.00 | 0.00 | 0.00 | 0.00 |
| *Propionibacterium* *acnes*\|*Propionibacterium* *acnes* KPA171202 | 0.01 | 0.06 | 0.00 | 0.00 | 0.00 | 0.00 | 0.00 |
| Order Pseudonocardiales | 0.02 | 0.35 | 0.00 | 0.00 | 0.00 | 0.00 | 0.00 |
| Family Pseudonocardiaceae | 0.02 | 0.35 | 0.00 | 0.00 | 0.00 | 0.00 | 0.00 |
| *Actinomycetospora* | 0.00 | 0.25 | 0.00 | 0.00 | 0.00 | 0.00 | 0.00 |
| *Actinomycetospora atypica* | 0.00 | 0.11 | 0.00 | 0.00 | 0.00 | 0.00 | 0.00 |
| *Actinomycetospora chiangmaiensis* | 0.00 | 0.13 | 0.00 | 0.00 | 0.00 | 0.00 | 0.00 |
| **Phylum Bacteroidetes** | 0.15 | 0.14 | 0.00 | 0.00 | 0.00 | 0.00 | 0.00 |
| **Class Sphingobacteriia** | 0.12 | 0.10 | 0.00 | 0.00 | 0.00 | 0.00 | 0.00 |
| Order Sphingobacteriales | 0.12 | 0.10 | 0.00 | 0.00 | 0.00 | 0.00 | 0.00 |
| Family Sphingobacteriaceae | 0.12 | 0.10 | 0.00 | 0.00 | 0.00 | 0.00 | 0.00 |
| *Mucilaginibacter* | 0.02 | 0.10 | 0.00 | 0.00 | 0.00 | 0.00 | 0.00 |
| *Mucilaginibacter koreensis* | 0.00 | 0.10 | 0.00 | 0.00 | 0.00 | 0.00 | 0.00 |
| *Nubsella* | 0.10 | 0.00 | 0.00 | 0.00 | 0.00 | 0.00 | 0.00 |
| *Nubsella zeaxanthinifaciens* | 0.10 | 0.00 | 0.00 | 0.00 | 0.00 | 0.00 | 0.00 |
| **Phylum Cyanobacteria/Melainabacteria group** | 45.11 | 4.15 | 25.24 | 24.60 | 1.53 | 32.90 | 21.95 |
| **Class Cyanobacteria** | 45.11 | 4.02 | 25.24 | 24.60 | 1.53 | 32.90 | 21.95 |
| Order Nostocales | 45.10 | 4.02 | 25.24 | 24.60 | 1.53 | 32.90 | 21.95 |
| Family Hapalosiphonaceae | 45.10 | 4.02 | 25.24 | 24.60 | 1.53 | 32.90 | 21.95 |
| *Fischerella* | 24.58 | 2.28 | 13.74 | 13.49 | 0.86 | 17.97 | 12.09 |
| *Fischerella muscicola*\|*Fischerella muscicola* PCC 7414 | 0.00 | 0.00 | 0.03 | 0.04 | 0.00 | 0.03 | 0.02 |
| *Fischerella thermalis* | 24.58 | 2.28 | 13.71 | 13.45 | 0.86 | 17.94 | 12.07 |
| *Mastigocoleus* | 20.52 | 1.74 | 11.50 | 11.11 | 0.67 | 14.93 | 9.86 |
| *Mastigocoleus testarum* | 20.52 | 1.74 | 11.50 | 11.11 | 0.67 | 14.93 | 9.86 |
| Class Cyanobacteria\|Oscillatoriophycideae | 0.00 | 0.13 | 0.00 | 0.00 | 0.00 | 0.00 | 0.00 |
| Order Oscillatoriales | 0.00 | 0.13 | 0.00 | 0.00 | 0.00 | 0.00 | 0.00 |
| Family Pseudanabaenaceae | 0.00 | 0.13 | 0.00 | 0.00 | 0.00 | 0.00 | 0.00 |
| *Tapinothrix* | 0.00 | 0.07 | 0.00 | 0.00 | 0.00 | 0.00 | 0.00 |
| *Tapinothrix clintonii*\|*Tapinothrix clintonii* GSE-PSE06-07G | 0.00 | 0.07 | 0.00 | 0.00 | 0.00 | 0.00 | 0.00 |
| **Phylum Firmicutes** | 0.04 | 0.00 | 0.03 | 0.02 | 0.00 | 0.02 | 0.01 |
| **Class Clostridia** | 0.04 | 0.00 | 0.03 | 0.02 | 0.00 | 0.02 | 0.01 |
| Order Clostridiales | 0.04 | 0.00 | 0.03 | 0.02 | 0.00 | 0.02 | 0.01 |
| Family Heliobacteriaceae | 0.04 | 0.00 | 0.03 | 0.02 | 0.00 | 0.02 | 0.01 |
| *Heliorestis* | 0.04 | 0.00 | 0.03 | 0.02 | 0.00 | 0.02 | 0.01 |
| *Heliorestis acidaminivorans* | 0.04 | 0.00 | 0.03 | 0.02 | 0.00 | 0.02 | 0.01 |
| **Phylum Proteobacteria** | 18.41 | 83.57 | 62.15 | 49.53 | 83.05 | 59.68 | 71.74 |
| **Class Alphaproteobacteria** | 2.78 | 7.79 | 4.83 | 5.90 | 0.48 | 3.21 | 2.25 |
| Order Caulobacterales | 0.01 | 0.13 | 0.00 | 0.00 | 0.00 | 0.00 | 0.00 |
| Family Caulobacteraceae | 0.01 | 0.13 | 0.00 | 0.00 | 0.00 | 0.00 | 0.00 |
| *Phenylobacterium* | 0.01 | 0.13 | 0.00 | 0.00 | 0.00 | 0.00 | 0.00 |
| *Phenylobacterium koreense* | 0.01 | 0.13 | 0.00 | 0.00 | 0.00 | 0.00 | 0.00 |
| Order Rhizobiales | 0.46 | 7.30 | 0.11 | 1.19 | 0.22 | 0.05 | 0.24 |
| Family Bradyrhizobiaceae | 0.12 | 0.24 | 0.01 | 0.01 | 0.00 | 0.00 | 0.00 |
| *Bradyrhizobium* | 0.10 | 0.14 | 0.01 | 0.00 | 0.00 | 0.00 | 0.00 |
| *Bradyrhizobium ottawaense* | 0.10 | 0.14 | 0.01 | 0.00 | 0.00 | 0.00 | 0.00 |
| *Salinarimonas* | 0.02 | 0.10 | 0.00 | 0.01 | 0.00 | 0.00 | 0.00 |
| *Salinarimonas rosea* | 0.02 | 0.10 | 0.00 | 0.01 | 0.00 | 0.00 | 0.00 |
| Family Methylobacteriaceae | 0.26 | 7.06 | 0.10 | 1.16 | 0.19 | 0.04 | 0.23 |
| *Methylobacterium* | 0.26 | 7.06 | 0.10 | 1.16 | 0.19 | 0.04 | 0.23 |
| *Methylobacterium phyllostachyos* | 0.12 | 6.76 | 0.09 | 1.13 | 0.12 | 0.03 | 0.11 |
| *Methylobacterium tarhaniae* | 0.06 | 0.11 | 0.01 | 0.03 | 0.07 | 0.01 | 0.12 |
| Family Rhizobiaceae | 0.04 | 0.00 | 0.00 | 0.01 | 0.03 | 0.00 | 0.00 |
| *Agrobacterium* | 0.04 | 0.00 | 0.00 | 0.01 | 0.03 | 0.00 | 0.00 |
| *Agrobacterium larrymoorei* | 0.04 | 0.00 | 0.00 | 0.01 | 0.03 | 0.00 | 0.00 |
| Order Rhodospirillales | 2.00 | 0.18 | 4.71 | 4.42 | 0.21 | 3.17 | 1.97 |
| Family Rhodospirillaceae | 2.00 | 0.18 | 4.71 | 4.42 | 0.21 | 3.17 | 1.97 |
| *Limimonas* | 2.00 | 0.18 | 4.71 | 4.42 | 0.21 | 3.17 | 1.97 |
| *Limimonas halophila* | 2.00 | 0.18 | 4.71 | 4.42 | 0.21 | 3.17 | 1.97 |
| Order Sphingomonadales | 0.25 | 0.17 | 0.01 | 0.30 | 0.04 | 0.00 | 0.04 |
| Family Sphingomonadaceae | 0.25 | 0.17 | 0.01 | 0.30 | 0.04 | 0.00 | 0.04 |
| *Sphingomonas* | 0.25 | 0.17 | 0.01 | 0.30 | 0.04 | 0.00 | 0.04 |
| *Sphingomonas dokdonensis* | 0.04 | 0.02 | 0.00 | 0.02 | 0.00 | 0.00 | 0.00 |
| *Sphingomonas kyungheensis* | 0.09 | 0.05 | 0.00 | 0.06 | 0.01 | 0.00 | 0.01 |
| *Sphingomonas paucimobilis* | 0.03 | 0.00 | 0.00 | 0.21 | 0.03 | 0.00 | 0.03 |
| **Class Betaproteobacteria** | 15.25 | 75.43 | 5.52 | 9.84 | 3.19 | 3.49 | 2.29 |
| Order Burkholderiales | 13.59 | 75.29 | 1.62 | 6.12 | 2.99 | 0.88 | 0.59 |
| Family Burkholderiaceae | 13.57 | 75.26 | 1.61 | 5.20 | 2.93 | 0.88 | 0.58 |
| *Burkholderia* | 13.57 | 74.99 | 1.61 | 5.20 | 2.93 | 0.88 | 0.58 |
| *Burkholderia cepacia* complex\|*Burkholderia lata* | 13.57 | 74.99 | 1.61 | 5.20 | 2.93 | 0.88 | 0.58 |
| *Caballeronia* | 0.00 | 0.26 | 0.00 | 0.00 | 0.00 | 0.00 | 0.00 |
| *Burkholderia megalochromosomata* | 0.00 | 0.26 | 0.00 | 0.00 | 0.00 | 0.00 | 0.00 |
| Family Oxalobacteraceae | 0.01 | 0.03 | 0.02 | 0.92 | 0.06 | 0.01 | 0.01 |
| *Massilia* | 0.01 | 0.03 | 0.02 | 0.92 | 0.06 | 0.01 | 0.01 |
| *Massilia consociata* | 0.01 | 0.03 | 0.02 | 0.92 | 0.06 | 0.01 | 0.01 |
| Order Neisseriales | 1.66 | 0.14 | 3.90 | 3.72 | 0.20 | 2.61 | 1.71 |
| Family Chromobacteriaceae | 1.66 | 0.14 | 3.90 | 3.72 | 0.20 | 2.61 | 1.71 |
| *Jeongeupia* | 1.66 | 0.14 | 3.90 | 3.72 | 0.20 | 2.61 | 1.71 |
| *Jeongeupia chitinilytica* | 1.66 | 0.14 | 3.90 | 3.72 | 0.20 | 2.61 | 1.71 |
| **Class Deltaproteobacteria** | 0.27 | 0.00 | 0.00 | 0.00 | 0.00 | 0.00 | 0.00 |
| Order Myxococcales | 0.27 | 0.00 | 0.00 | 0.00 | 0.00 | 0.00 | 0.00 |
| Family Cystobacterineae | 0.27 | 0.00 | 0.00 | 0.00 | 0.00 | 0.00 | 0.00 |
| *Cystobacteraceae* | 0.27 | 0.00 | 0.00 | 0.00 | 0.00 | 0.00 | 0.00 |
| *Cystobacter*\|*Cystobacter velatus* | 0.27 | 0.00 | 0.00 | 0.00 | 0.00 | 0.00 | 0.00 |
| **Class Gammaproteobacteria** | 0.11 | 0.35 | 51.81 | 33.79 | 79.39 | 52.98 | 67.20 |
| Order Alteromonadales | 0.00 | 0.00 | 0.06 | 0.05 | 0.00 | 0.02 | 0.01 |
| Family Alteromonadaceae | 0.00 | 0.00 | 0.06 | 0.05 | 0.00 | 0.02 | 0.01 |
| *Aliagarivorans* | 0.00 | 0.00 | 0.06 | 0.05 | 0.00 | 0.02 | 0.01 |
| *Aliagarivorans marinus* | 0.00 | 0.00 | 0.06 | 0.05 | 0.00 | 0.02 | 0.01 |
| Order Enterobacteriales | 0.05 | 0.03 | 51.61 | 33.65 | 77.99 | 52.76 | 66.50 |
| Family Enterobacteriaceae | 0.05 | 0.03 | 51.61 | 33.65 | 77.99 | 52.76 | 66.50 |
| *Enterobacter* | 0.00 | 0.00 | 0.00 | 0.01 | 0.02 | 0.27 | 0.30 |
| *Enterobacter aerogenes*\|*Enterobacter aerogenes* KCTC 2190 | 0.00 | 0.00 | 0.00 | 0.01 | 0.02 | 0.27 | 0.30 |
| *Erwinia* | 0.00 | 0.00 | 0.01 | 0.01 | 0.74 | 0.10 | 0.16 |
| *Erwinia toletana* | 0.00 | 0.00 | 0.01 | 0.01 | 0.74 | 0.10 | 0.16 |
| *Hafnia* | 0.00 | 0.00 | 0.02 | 0.03 | 0.41 | 0.27 | 0.31 |
| *Hafnia alvei* | 0.00 | 0.00 | 0.02 | 0.03 | 0.40 | 0.15 | 0.23 |
| *Hafnia paralvei* | 0.00 | 0.00 | 0.00 | 0.00 | 0.01 | 0.12 | 0.08 |
| *Kluyvera* | 0.00 | 0.01 | 0.04 | 0.06 | 0.15 | 20.29 | 19.26 |
| *Kluyvera cryocrescens* | 0.00 | 0.01 | 0.04 | 0.06 | 0.15 | 20.29 | 19.26 |
| *Pantoea* | 0.05 | 0.01 | 0.41 | 1.04 | 51.29 | 23.66 | 35.67 |
| *Pantoea brenneri* | 0.00 | 0.00 | 0.00 | 0.00 | 0.09 | 0.02 | 0.03 |
| *Pantoea dispersa* | 0.00 | 0.00 | 0.00 | 0.01 | 0.00 | 0.01 | 0.05 |
| *Pantoea eucrina* | 0.02 | 0.00 | 0.02 | 0.13 | 0.08 | 0.14 | 1.03 |
| *Pantoea septica* | 0.03 | 0.01 | 0.39 | 0.90 | 51.12 | 23.50 | 34.56 |
| *Pseudocitrobacter* | 0.00 | 0.01 | 0.06 | 0.10 | 0.00 | 0.05 | 0.24 |
| *Pseudocitrobacter anthropi* | 0.00 | 0.01 | 0.06 | 0.10 | 0.00 | 0.05 | 0.24 |
| *Raoultella* | 0.00 | 0.00 | 0.00 | 0.00 | 0.00 | 0.21 | 0.10 |
| *Raoultella planticola*\|*Raoultella planticola* ATCC 33531 | 0.00 | 0.00 | 0.00 | 0.00 | 0.00 | 0.21 | 0.10 |
| *Rosenbergiella* | 0.00 | 0.00 | 0.04 | 0.02 | 0.44 | 0.21 | 0.29 |
| *Rosenbergiella collisarenosi* | 0.00 | 0.00 | 0.00 | 0.00 | 0.04 | 0.01 | 0.02 |
| *Rosenbergiella epipactidis* | 0.00 | 0.00 | 0.03 | 0.02 | 0.40 | 0.20 | 0.27 |
| unclassified Enterobacteriaceae | 0.00 | 0.00 | 51.03 | 32.38 | 24.93 | 7.70 | 10.19 |
| ant, tsetse, mealybug, aphid, etc. endosymbionts\|aphid secondary symbionts\|secondary endosymbiont of *Heteropsylla cubana* | 0.00 | 0.00 | 51.03 | 32.38 | 24.93 | 7.70 | 10.19 |
| Order Oceanospirillales | 0.07 | 0.34 | 0.01 | 0.01 | 0.01 | 0.01 | 0.01 |
| Family Halomonadaceae | 0.07 | 0.34 | 0.01 | 0.01 | 0.01 | 0.01 | 0.01 |
| *Halomonas* | 0.07 | 0.34 | 0.01 | 0.01 | 0.01 | 0.01 | 0.01 |
| *Halomonas stevensii*\|*Halomonas stevensii* S18214 | 0.07 | 0.34 | 0.01 | 0.01 | 0.01 | 0.01 | 0.01 |
| Order Pseudomonadales | 0.00 | 0.00 | 0.01 | 0.04 | 1.39 | 0.16 | 0.68 |
| Family Pseudomonadaceae | 0.00 | 0.00 | 0.01 | 0.04 | 1.39 | 0.16 | 0.68 |
| *Pseudomonas* | 0.00 | 0.00 | 0.01 | 0.04 | 1.39 | 0.16 | 0.68 |
| *Pseudomonas putida* group\|*Pseudomonas oryzihabitans* | 0.00 | 0.00 | 0.01 | 0.04 | 1.39 | 0.16 | 0.68 |
| Order Vibrionales | 0.00 | 0.00 | 0.11 | 0.04 | 0.00 | 0.02 | 0.01 |
| Family Vibrionaceae | 0.00 | 0.00 | 0.11 | 0.04 | 0.00 | 0.02 | 0.01 |
| *Photobacterium* | 0.00 | 0.00 | 0.11 | 0.04 | 0.00 | 0.02 | 0.01 |
| *Photobacterium leiognathi*\|*Photobacterium leiognathi* subsp. *mandapamensis* | 0.00 | 0.00 | 0.11 | 0.04 | 0.00 | 0.02 | 0.01 |
| No blast hit | 36.18 | 11.10 | 12.57 | 25.83 | 15.40 | 7.40 | 6.30 |
