# Supplementary material for: Core members and differential abundance of chrysomelid microbiota in the life stages of Podontiaaffinis (Galerucinae) and adult Silanafarinosa (Cassidinae, Coleoptera)
Source: Biodivers Data J. 2022 Oct 7;10:e87459. doi: 10.3897/BDJ.10.e87459 (PMC9836631; doi:10.3897/BDJ.10.e87459)
Supplement: Supplementary material 4 — Bacterial OTUs detected in Podontiaaffinis female adults (PA), P.affinis larvae (PAL) and Salina farinosa (SF) female adults [file bdj-10-e87459-s004.docx]

**Table S4**

Bacterial OTUs detected in *Podontia affinis* female adults (PA), *P. affinis* larvae (PAL), and *Salina farinosa* (SF) female adults. +, present; –, absent.

| Bacterial OTU | PA | PAL | SF |
| --- | --- | --- | --- |
| **Phylum** |  |  |  |
| Acidobacteria | + | + | – |
| Actinobacteria | + | + | + |
| Bacteroidetes | + | + | + |
| Cyanobacteria/Melainabacteria group | + | + | + |
| Deinococcus-Thermus | + | + | – |
| Firmicutes | + | + | + |
| Planctomycetes | + | + | – |
| Proteobacteria | + | + | + |
| **Class** |  |  |  |
| Acidobacteriia | + | + | – |
| Actinobacteria | + | + | + |
| Cytophagia | + | + | – |
| Flavobacteriia | – | + | – |
| Sphingobacteriia | + | + | + |
| Cyanobacteria | + | + | + |
| Cyanobacteria/Oscillatoriophycideae | + | – | + |
| Deinococci | + | + | – |
| Clostridia | + | + | + |
| Planctomycetia | + | + | – |
| Alphaproteobacteria | + | + | + |
| Betaproteobacteria | + | + | + |
| Deltaproteobacteria | + | + | + |
| Gammaproteobacteria | + | + | + |
| **Order** |  |  |  |
| Acidobacteriales | + | + | – |
| Frankiales | + | – | – |
| Kineosporiales | + | + | – |
| Micrococcales | + | + | + |
| Micromonosporales | + | + | – |
| Propionibacteriales | + | + | + |
| Pseudonocardiales | + | + | + |
| Cytophagales | + | + | – |
| Flavobacteriales | – | + | – |
| Sphingobacteriales | + | + | + |
| Pleurocapsales | + | + | – |
| Nostocales | + | + | + |
| Oscillatoriales | + | – | + |
| Deinococcales | + | + | – |
| Clostridiales | + | + | + |
| Planctomycetales | + | + | – |
| Caulobacterales | + | + | – |
| Rhizobiales | + | + | + |
| Rhodobacterales | + | + | – |
| Rhodospirillales | + | + | + |
| Sphingomonadales | + | + | + |
| Burkholderiales | + | + | + |
| Neisseriales | + | + | + |
| Myxococcales | + | + | + |
| Alteromonadales | – | – | + |
| Enterobacteriales | + | – | + |
| Oceanospirillales | + | + | + |
| Pseudomonadales | + | + | + |
| Vibtionales | – | – | + |
| Xanthomonadales | + | – | – |
| **Family** |  |  |  |
| Acidobacteriaceae | + | + | – |
| Frankiaceae | + | – | – |
| Kineosporaceae | + | + | – |
| Microbacteriaceae | + | + | + |
| Micrococcaceae | + | + | – |
| Micromonosporaceae | + | + | – |
| Propionibacteriaceae | + | + | + |
| Pseudonocardiaceae | + | + | + |
| Cytophagaceae | + | + | – |
| Flavobacteriaceae | – | + | – |
| Sphingobacteriaceae | + | + | + |
| Chroococcidiopsidaceae | + | + | – |
| Hapalosiphonaceae | + | + | + |
| Oscillatoriaceae | + | – | + |
| Deinococcaceae | + | + | – |
| Heliobacteriaceae | + | + | + |
| Isosphaeraceae | + | + | – |
| Caulobacteriaceae | + | + | – |
| Aurantimonadaceae | + | + | – |
| Bradyrhizobiaceae | + | + | + |
| Brucellaceae | + | + | – |
| Methylobacteriaceae | + | + | + |
| Rhizobiaceae | + | + | + |
| Rhodobacteraceae | + | + | – |
| Acetobacteraceae | + | + | – |
| Rhodospirillaceae | + | + | + |
| Sphingomonadaceae | + | + | + |
| Burkholderiaceae | + | + | + |
| Comamonadaceae | + | + | – |
| Oxalobacteraceae | + | + | + |
| Chromobacteriaceae | + | + | + |
| Cystobacteraceae | + | + | + |
| Alteromonadaceae | – | – | + |
| Enterobacteriaceae | – | – | + |
| Halomonadaceae | + | + | + |
| Moraxellaceae | + | + | – |
| Pseudomonadaceae | + | + | + |
| Vibrionaceae | – | – | + |
| Xanthomonadaceae | + | – | – |
| **Genus** |  |  |  |
| *Terriglobus* | + | + | – |
| *Jatrophihabitans* | + | – | – |
| *Kineococcus* | + | + | – |
| *Curtobacterium* | + | + | + |
| *Kocuria* | + | + | – |
| *Catellatospora* | + | + | – |
| *Leifsonia* | + | – | + |
| *Propionibacterium* | + | + | + |
| *Actinomycetospora* | + | + | + |
| *Pseudonocardia* | + | – | – |
| *Hymenobacter* | + | + | – |
| *Spirosoma* | – | + | – |
| *Chryseobacterium* | – | + | – |
| *Mucilaginibacter* | + | + | + |
| *Nubsella* | + | – | – |
| *Chroococcidiopsis* | + | + | – |
| *Fischerella* | + | + | + |
| *Mastigocoleus* | + | + | + |
| *Aerosakkonema* | + | – | – |
| *Tapinothrix* | + | – | + |
| *Deinococcus* | + | + | – |
| *Heliorestis* | + | + | + |
| *Aquisphaera* | + | + | – |
| *Phenylobacterium* | + | + | – |
| *Aureimonas* | + | + | – |
| *Bradyrhizobium* | + | + | + |
| *Salinarimonas* | + | + | + |
| *Mycoplana* | + | + | – |
| *Methylobacterium* | + | + | + |
| *Agrobacterium* | + | + | + |
| *Paracoccus* | + | – | – |
| *Rosiebium* | + | + | – |
| *Roseomonas* | + | + | – |
| *Limimonas* | + | + | + |
| *Sphingomonas* | + | + | + |
| *Burkholderia* | + | + | + |
| *Caballeronia* | + | + | – |
| *Ramlibacter* | + | + | – |
| *Massilia* | + | + | + |
| *Jeogeupia* | + | + | + |
| Cystobacteraceae | + | + | + |
| *Aliagarivorans* | – | – | + |
| *Enterobacter* | – | – | + |
| *Erwinia* | – | – | + |
| *Hafnia* | – | – | + |
| *Kluyvera* | + | – | + |
| *Pantoea* | + | – | + |
| *Pseudocitrobacter* | + | – | + |
| *Raoultella* | – | – | + |
| *Rosenbergiella* | – | – | + |
| *Halomonas* | + | + | + |
| *Moraxella* | + | + | – |
| *Pseudomonas* | + | + | + |
| *Photobacterium* | – | – | + |
| *Stenotrophomonas* | + | – | – |
| **Species** |  |  |  |
| *Terriglobus tenax* | + | + | – |
| *Jatrophihabitans endophyticus* | + | – | – |
| *Kineococcus endophyticus* | + | + | – |
| *Curtobacterium oceanosedimentum* | + | + | + |
| *Kocuria palustris* | + | + | – |
| *Catellatospora coxensis* | + | + | – |
| *Leifsonia shinshuensis* | + | – | + |
| *Propionibacterium acnes* | + | + | + |
| *Actinomycetospora atypica* | + | – | + |
| *Actinomycetospora chiangmaiensis* | + | + | + |
| *Pseudonocardia ammonioxydans* | + | – | – |
| *Pseudonocardia kujensis* | + | – | – |
| *Hymenobacter aerophilus* | – | + | – |
| *Hymenobacter flocculans* | + | + | – |
| *Hymenobacter metalli* | + | + | – |
| *Hymenobacter ocellatus* | + | + | – |
| *Spirosoma oryzae* | – | + | – |
| *Chryseobacterium* | – | + | – |
| *Mucilaginibacter daejeonensis* | + | + | – |
| *Mucilaginibacter korensis* | + | + | + |
| *Mucilaginibacter lutimaris* | + | + | – |
| *Nubsella zeaxanthinifaciens* | + | – | – |
| *Chroococcidiopsis thermalis* | + | + | – |
| *Fischerella muscicola* | – | – | + |
| *Fischerella thermalis* | + | + | + |
| *Mustigocoleus testarum* | + | + | + |
| *Aerosakkonema funiforme* | + | – | – |
| *Tapinothrix clintonii* | + | – | + |
| *Deinococcus xinjiangensis* | + | + | – |
| *Heliorestis acidaminivorans* | + | + | + |
| *Aquisphaera giovannonii* | + | + | – |
| *Phenylobacterium koreense* | + | + | – |
| *Aureimonas ureilytica* | + | + | – |
| *Bradyrhizobium ottawaense* | + | + | + |
| *Salinarimonas rosea* | + | + | + |
| *Mycoplana ramosa* | + | + | – |
| *Methylobacterium aerolatum* | + | + | – |
| *Methylobacterium extorquens* | + | + | – |
| *Methylobacterium iners* | + | – | – |
| *Methylobacterium komagatae* | + | + | – |
| *Methylobacterium phyllostachyos* | + | + | + |
| *Methylobacterium radiotolerans* | + | + | – |
| *Methylobacterium tarhaniae* | + | + | + |
| *Agrobacterium larrymoorei* | + | + | + |
| *Paracoccus aminovorans* | + | – | – |
| *Roseibium aquae* | + | + | – |
| *Roseomonas aerilata* | + | + | – |
| *Limimonas halophila* | + | + | + |
| *Sphingomonas canadensis* | + | + | – |
| *Sphingomonas changbaiensis* | + | + | – |
| *Sphingomonas dokdonensis* | + | – | + |
| *Sphingomonas echinoides* | + | + | – |
| *Sphingomonas endophytica* | + | + | – |
| *Sphingomonas guangdongensis* | – | + | – |
| *Sphingomonas kyungheensis* | + | + | + |
| *Sphingomonas paucimobilis* | + | + | + |
| *Sphingomonas roseiflava* | + | + | – |
| *Sphingomonas wittichii* | + | + | – |
| *Sphingomonas yunnanensis* | + | + | – |
| *Burkholdera lata* | + | + | + |
| *Burkholderia megalochromosomata* | + | + | – |
| *Ramlibacter solisilvae* | + | + | – |
| *Massilia consociata* | + | + | + |
| *Jeongeupia chitinilytica* | + | + | + |
| *Cystobacter velatus* | + | + | + |
| *Aliagarivorans marinus* | – | – | + |
| *Enterobacter aerogenes* | – | – | + |
| *Erwinia toletana* | – | – | + |
| *Hafnia alvei* | – | – | + |
| *Hafnia paralvei* | – | – | + |
| *Kluyvera cryocrescens* | + | – | + |
| *Pantoea brenneri* | – | – | + |
| *Pantoea dispersa* | – | – | + |
| *Pantoea eucrina* | + | – | + |
| *Pantoea septica* | + | – | + |
| *Pseudocitrobacter anthropi* | + | – | + |
| *Raoultella planticola* | – | – | + |
| *Rosenbergiella collisarenosi* | – | – | + |
| *Rosenbergiella epipactidis* | – | – | + |
| Secondary symbiont of *Heteropsylla cubana* | + | – | + |
| *Halomonas stevensii* | + | + | + |
| *Moraxella osloensis* | + | + | – |
| *Pseudomonas oryzihabitans* | + | + | + |
| *Photobacterium leiognathi* | – | – | + |
| *Stenotrophomonas maltophilia* | + | – | – |
| Total | 198 | 170 | 126 |
