# Supplementary material for: Core members and differential abundance of chrysomelid microbiota in the life stages of Podontiaaffinis (Galerucinae) and adult Silanafarinosa (Cassidinae, Coleoptera)
Source: Biodivers Data J. 2022 Oct 7;10:e87459. doi: 10.3897/BDJ.10.e87459 (PMC9836631; doi:10.3897/BDJ.10.e87459)
Supplement: Supplementary material 5 — Comparisons of relative abundance of bacterial OTUs between the larvae and adult beetles of Podontiaaffinis [file bdj-10-e87459-s005.docx]

**Table S5**

Comparisons of relative abundance of bacterial OTUs between the larvae and adult beetles of *Podontia affinis*. *indicates significantly different.

|  | PA2 & PA3  Mean±SD | PAL4-PAL6  Mean±SD | ANOVA F-stat | p-value |
| --- | --- | --- | --- | --- |
| **Phylum Acidobacteria** | 0.06±0.06 | 0±0 | 3.82 | 0.15 |
| **Class Acidobacteriia** | 0.06±0.06 | 0±0 | 3.82 | 0.15 |
| Order Acidobacteriales | 0.06±0.06 | 0±0 | 3.82 | 0.15 |
| Family Acidobacteriaceae | 0.06±0.06 | 0±0 | 3.82 | 0.15 |
| *Terriglobus* | 0.06±0.06 | 0±0 | 3.82 | 0.15 |
| *Terriglobus tenax* | 0.06±0.06 | 0±0 | 3.82 | 0.15 |
| **Phylum Actinobacteria** | 0.51±0.58 | 0.33±0.06 | 0.34 | 0.60 |
| **Class Actinobacteria** | 0.51±0.58 | 0.33±0.06 | 0.34 | 0.60 |
| Order Frankiales | 0.03±0.03 | 0±0 | 1.80 | 0.27 |
| Family Frankiaceae | 0.03±0.03 | 0±0 | 1.80 | 0.27 |
| *Jatrophihabitans* | 0.03±0.03 | 0±0 | 1.80 | 0.27 |
| *Jatrophihabitans endophyticus* | 0.03±0.03 | 0±0 | 1.80 | 0.27 |
| Order Kineosporiales | 0±0 | 0.05±0.02 | 11.25 | 0.04 |
| Family Kineosporiaceae | 0±0 | 0.05±0.02 | 11.25 | 0.04 |
| *Kineococcus* | 0±0 | 0.05±0.02 | 11.25 | 0.04 |
| *Kineococcus endophyticus* | 0±0 | 0.05±0.02 | 11.25 | 0.04 |
| Order Micrococcales | 0.26±0.29 | 0.26±0.05 | 0.00 | 0.98 |
| Family Microbacteriaceae | 0.23±0.29 | 0.25±0.05 | 0.02 | 0.89 |
| *Curtobacterium* | 0.23±0.29 | 0.25±0.05 | 0.02 | 0.89 |
| *Curtobacterium oceanosedimentum* | 0.23±0.29 | 0.25±0.05 | 0.02 | 0.89 |
| Family Micrococcaceae | 0.03±0.01 | 0.01±0 | 30.94 | 0.01* |
| *Kocuria* | 0.03±0.01 | 0.01±0 | 30.94 | 0.01* |
| *Kocuria palustris* | 0.03±0.01 | 0.01±0 | 30.94 | 0.01* |
| Order Micromonosporales | 0±0 | 0.02±0.02 | 1.15 | 0.36 |
| Family Micromonosporaceae | 0±0 | 0.02±0.02 | 1.15 | 0.36 |
| *Catellatospora* | 0±0 | 0.02±0.02 | 1.15 | 0.36 |
| *Catellatospora coxensis* | 0±0 | 0.02±0.02 | 1.15 | 0.36 |
| Order Propionibacteriales | 0.03±0.04 | 0±0 | 3.53 | 0.16 |
| Family Propionibacteriaceae | 0.03±0.04 | 0±0 | 3.53 | 0.16 |
| *Propionibacterium* | 0.03±0.04 | 0±0 | 3.53 | 0.16 |
| *Propionibacterium acnes*\|*Propionibacterium acnes* KPA171202 | 0.03±0.04 | 0±0 | 3.53 | 0.16 |
| Order Pseudonocardiales | 0.19±0.23 | 0±0 | 2.26 | 0.23 |
| Family Pseudonocardiaceae | 0.19±0.23 | 0±0 | 2.26 | 0.23 |
| *Actinomycetospora* | 0.13±0.17 | 0±0 | 1.80 | 0.27 |
| *Actinomycetospora atypica* | 0.06±0.08 | 0±0 | 1.80 | 0.27 |
| *Actinomycetospora chiangmaiensis* | 0.07±0.09 | 0±0 | 1.80 | 0.27 |
| *Pseudonocardia* | 0.06±0.06 | 0±0 | 3.76 | 0.15 |
| *Pseudonocardia ammonioxydans* | 0.03±0.03 | 0±0 | 3.53 | 0.16 |
| *Pseudonocardia kujensis* | 0.03±0.03 | 0±0 | 4.05 | 0.14 |
| **Phylum Bacteroidetes** | 0.14±0.01 | 1.4±1.36 | 1.54 | 0.30 |
| **Class Cytophagia** | 0.03±0.01 | 1.26±1.31 | 1.58 | 0.30 |
| Order Cytophagales | 0.03±0.01 | 1.26±1.31 | 1.58 | 0.30 |
| Family Cytophagaceae | 0.03±0.01 | 1.26±1.31 | 1.58 | 0.30 |
| *Hymenobacter* | 0.03±0.01 | 0.88±0.81 | 2.01 | 0.25 |
| *Hymenobacter aerophilus* | 0±0 | 0.26±0.44 | 0.62 | 0.49 |
| *Hymenobacter flocculans* | 0±0.01 | 0.22±0.37 | 0.60 | 0.50 |
| *Hymenobacter metalli* | 0.02±0.02 | 0.37±0.25 | 3.47 | 0.16 |
| *Hymenobacter ocellatus* | 0±0.01 | 0.03±0.04 | 0.59 | 0.50 |
| *Spirosoma* | 0±0 | 0.38±0.51 | 1.01 | 0.39 |
| *Spirosoma oryzae* | 0±0 | 0.38±0.51 | 1.01 | 0.39 |
| **Class Flavobacteriia** | 0±0 | 0.02±0.03 | 0.60 | 0.50 |
| Order Flavobacteriales | 0±0 | 0.02±0.03 | 0.60 | 0.50 |
| Family Flavobacteriaceae | 0±0 | 0.02±0.03 | 0.60 | 0.50 |
| *Chryseobacterium* | 0±0 | 0.02±0.03 | 0.60 | 0.50 |
| *Chryseobacterium gambrini* | 0±0 | 0.02±0.03 | 0.60 | 0.50 |
| **Class Sphingobacteriia** | 0.11±0.01 | 0.13±0.1 | 0.08 | 0.80 |
| Order Sphingobacteriales | 0.11±0.01 | 0.13±0.1 | 0.08 | 0.80 |
| Family Sphingobacteriaceae | 0.11±0.01 | 0.13±0.1 | 0.08 | 0.80 |
| *Mucilaginibacter* | 0.06±0.06 | 0.13±0.1 | 0.81 | 0.43 |
| *Mucilaginibacter daejeonensis* | 0±0 | 0.04±0.06 | 0.78 | 0.44 |
| *Mucilaginibacter koreensis* | 0.05±0.07 | 0.02±0.02 | 0.58 | 0.50 |
| *Mucilaginibacter lutimaris* | 0.01±0.01 | 0.07±0.06 | 2.30 | 0.23 |
| *Nubsella* | 0.05±0.07 | 0±0 | 1.80 | 0.27 |
| *Nubsella zeaxanthinifaciens* | 0.05±0.07 | 0±0 | 1.80 | 0.27 |
| **Phylum Cyanobacteria/Melainabacteria group** | 24.63±28.96 | 58.2±3.85 | 4.67 | 0.12 |
| **Class Cyanobacteria** | 24.57±29.05 | 58.2±3.85 | 4.66 | 0.12 |
| Order Pleurocapsales | 0±0 | 0.02±0 | 15.00 | 0.03* |
| Family Chroococcidiopsidaceae | 0±0 | 0.02±0 | 15.00 | 0.03* |
| *Chroococcidiopsis* | 0±0 | 0.02±0 | 15.00 | 0.03* |
| *Chroococcidiopsis thermalis*\|*Chroococcidiopsis thermalis* PCC 7203 | 0±0 | 0.02±0 | 15.00 | 0.03* |
| Order Stigonematales | 24.56±29.05 | 58.19±3.85 | 4.66 | 0.12 |
| Family Stigonemataceae | 24.56±29.05 | 58.19±3.85 | 4.66 | 0.12 |
| *Fischerella* | 13.43±15.77 | 31.67±2.27 | 4.63 | 0.12 |
| *Fischerella thermalis* | 13.43±15.77 | 31.67±2.27 | 4.63 | 0.12 |
| *Mastigocoleus* | 11.13±13.28 | 26.51±1.6 | 4.70 | 0.12 |
| *Mastigocoleus testarum* | 11.13±13.28 | 26.51±1.6 | 4.70 | 0.12 |
| **Class Cyanobacteria\|Oscillatoriophycideae** | 0.06±0.09 | 0±0 | 1.80 | 0.27 |
| Order Oscillatoriales | 0.06±0.09 | 0±0 | 1.80 | 0.27 |
| Family Pseudanabaenaceae | 0.06±0.09 | 0±0 | 1.80 | 0.27 |
| *Aerosakkonema* | 0.03±0.04 | 0±0 | 1.80 | 0.27 |
| *Aerosakkonema funiforme* | 0.03±0.04 | 0±0 | 1.80 | 0.27 |
| *Tapinothrix* | 0.04±0.05 | 0±0 | 1.80 | 0.27 |
| *Tapinothrix clintonii*\|*Tapinothrix clintonii* GSE-PSE06-07G | 0.04±0.05 | 0±0 | 1.80 | 0.27 |
| **Phylum Deinococcus-Thermus** | 0.01±0.02 | 0.01±0.02 | 0.00 | 1.00 |
| **Class Deinococci** | 0.01±0.02 | 0.01±0.02 | 0.00 | 1.00 |
| Order Deinococcales | 0.01±0.02 | 0.01±0.02 | 0.00 | 1.00 |
| Family Deinococcaceae | 0.01±0.02 | 0.01±0.02 | 0.00 | 1.00 |
| *Deinococcus* | 0.01±0.02 | 0.01±0.02 | 0.00 | 1.00 |
| *Deinococcus xinjiangensis* | 0.01±0.02 | 0.01±0.02 | 0.00 | 1.00 |
| **Phylum Firmicutes** | 0.02±0.03 | 0.19±0.09 | 6.27 | 0.09 |
| **Class Clostridia** | 0.02±0.03 | 0.19±0.09 | 6.27 | 0.09 |
| Order Clostridiales | 0.02±0.03 | 0.19±0.09 | 6.27 | 0.09 |
| Family Heliobacteriaceae | 0.02±0.03 | 0.19±0.09 | 6.27 | 0.09 |
| *Heliorestis* | 0.02±0.03 | 0.19±0.09 | 6.27 | 0.09 |
| *Heliorestis acidaminivorans* | 0.02±0.03 | 0.19±0.09 | 6.27 | 0.09 |
| **Phylum Planctomycetes** | 0.01±0 | 0.13±0.08 | 4.59 | 0.12 |
| **Class Planctomycetia** | 0.01±0 | 0.13±0.08 | 4.59 | 0.12 |
| Order Planctomycetales | 0.01±0 | 0.13±0.08 | 4.59 | 0.12 |
| Family Isosphaeraceae | 0.01±0 | 0.13±0.08 | 4.59 | 0.12 |
| *Aquisphaera* | 0.01±0 | 0.13±0.08 | 4.59 | 0.12 |
| *Aquisphaera giovannonii* | 0.01±0 | 0.13±0.08 | 4.59 | 0.12 |
| **Phylum Proteobacteria** | 50.99±46.08 | 29.24±3.92 | 0.79 | 0.44 |
| **Class Alphaproteobacteria** | 5.28±3.54 | 16.23±3.99 | 9.70 | 0.05 |
| Order Caulobacterales | 0.07±0.09 | 0±0 | 2.45 | 0.22 |
| Family Caulobacteraceae | 0.07±0.09 | 0±0 | 2.45 | 0.22 |
| *Phenylobacterium* | 0.07±0.09 | 0±0 | 2.45 | 0.22 |
| *Phenylobacterium koreense* | 0.07±0.09 | 0±0 | 2.45 | 0.22 |
| Order Rhizobiales | 3.88±4.84 | 3.92±1 | 0.00 | 0.99 |
| Family Aurantimonadaceae | 0±0.01 | 0.52±0.69 | 1.03 | 0.39 |
| *Aureimonas* | 0±0.01 | 0.52±0.69 | 1.03 | 0.39 |
| *Aureimonas ureilytica*\|*Aureimonas ureilytica* DSM 18598 = NBRC 106430 | 0±0.01 | 0.52±0.69 | 1.03 | 0.39 |
| Family Bradyrhizobiaceae | 0.18±0.08 | 0.2±0.25 | 0.01 | 0.91 |
| *Bradyrhizobium* | 0.12±0.03 | 0.01±0.01 | 56.54 | 0.00* |
| *Bradyrhizobium ottawaense* | 0.12±0.03 | 0.01±0.01 | 56.54 | 0.00* |
| *Salinarimonas* | 0.06±0.05 | 0.2±0.24 | 0.56 | 0.51 |
| *Salinarimonas rosea* | 0.06±0.05 | 0.2±0.24 | 0.56 | 0.51 |
| Family Brucellaceae | 0.01±0.01 | 0.04±0.05 | 0.73 | 0.46 |
| *Mycoplana* | 0.01±0.01 | 0.04±0.05 | 0.73 | 0.46 |
| *Mycoplana ramosa* | 0.01±0.01 | 0.04±0.05 | 0.73 | 0.46 |
| Family Methylobacteriaceae | 3.66±4.81 | 3.01±0.85 | 0.06 | 0.82 |
| *Methylobacterium* | 3.66±4.81 | 3.01±0.85 | 0.06 | 0.82 |
| *Methylobacterium aerolatum* | 0.02±0.02 | 0.05±0.03 | 2.10 | 0.24 |
| *Methylobacterium extorquens* group\|*Methylobacterium extorquens*\|*Methylobacterium extorquens* AM1 | 0.02±0.02 | 0.06±0.06 | 0.95 | 0.40 |
| *Methylobacterium iners* | 0.03±0.03 | 0±0 | 1.80 | 0.27 |
| *Methylobacterium komagatae*\|*Methylobacterium komagatae* DSM 19563 | 0.05±0 | 0.26±0.41 | 0.46 | 0.55 |
| *Methylobacterium phyllostachyos* | 3.44±4.7 | 2.1±0.41 | 0.29 | 0.63 |
| *Methylobacterium radiotolerans*\|*Methylobacterium radiotolerans* JCM 2831 | 0.03±0.04 | 0.06±0.05 | 0.58 | 0.50 |
| *Methylobacterium tarhaniae* | 0.08±0.04 | 0.49±0.46 | 1.37 | 0.33 |
| Family Rhizobiaceae | 0.02±0.03 | 0.14±0.15 | 1.03 | 0.39 |
| *Agrobacterium* | 0.02±0.03 | 0.14±0.15 | 1.03 | 0.39 |
| *Agrobacterium larrymoorei* | 0.02±0.03 | 0.14±0.15 | 1.03 | 0.39 |
| Order Rhodobacterales | 0.03±0.04 | 0.02±0 | 0.20 | 0.69 |
| Family Rhodobacteraceae | 0.03±0.04 | 0.02±0 | 0.20 | 0.69 |
| *Paracoccus* | 0.03±0.04 | 0±0 | 1.80 | 0.27 |
| *Paracoccus aminovorans* | 0.03±0.04 | 0±0 | 1.80 | 0.27 |
| *Roseibium* | 0±0 | 0.02±0 | 438.00 | 0.00* |
| *Roseibium aquae* | 0±0 | 0.02±0 | 438.00 | 0.00* |
| Order Rhodospirillales | 1.09±1.29 | 7.79±2.37 | 12.56 | 0.04* |
| Family Acetobacteraceae | 0±0.01 | 0.05±0.03 | 3.94 | 0.14 |
| *Roseomonas* | 0±0.01 | 0.05±0.03 | 3.94 | 0.14 |
| *Roseomonas aerilata* | 0±0.01 | 0.05±0.03 | 3.94 | 0.14 |
| Family Rhodospirillaceae | 1.09±1.29 | 7.74±2.39 | 12.16 | 0.04* |
| *Limimonas* | 1.09±1.29 | 7.74±2.39 | 12.16 | 0.04* |
| *Limimonas halophila* | 1.09±1.29 | 7.74±2.39 | 12.16 | 0.04* |
| Order Sphingomonadales | 0.21±0.06 | 4.5±4.89 | 1.39 | 0.32 |
| Family Sphingomonadaceae | 0.21±0.06 | 4.5±4.89 | 1.39 | 0.32 |
| *Sphingomonas* | 0.21±0.06 | 4.5±4.89 | 1.39 | 0.32 |
| *Sphingomonas canadensis* | 0.01±0.01 | 0.12±0.1 | 2.04 | 0.25 |
| *Sphingomonas changbaiensis*\|*Sphingomonas changbaiensis* NBRC 104936 | 0.01±0 | 0.06±0.04 | 2.26 | 0.23 |
| *Sphingomonas echinoides* | 0.01±0 | 0.01±0.01 | 0.60 | 0.50 |
| *Sphingomonas endophytica* | 0.03±0.02 | 2.19±2.76 | 1.11 | 0.37 |
| *Sphingomonas guangdongensis* | 0±0 | 0.02±0.01 | 7.35 | 0.07 |
| *Sphingomonas kyungheensis* | 0.07±0.03 | 0.62±0.45 | 2.58 | 0.21 |
| *Sphingomonas paucimobilis* | 0.02±0.02 | 1.01±1.32 | 1.02 | 0.39 |
| *Sphingomonas roseiflava* | 0.03±0.01 | 0.4±0.37 | 1.78 | 0.27 |
| *Sphingomonas wittichii*\|*Sphingomonas wittichii* RW1 | 0.04±0.03 | 0.04±0.03 | 0.05 | 0.84 |
| *Sphingomonas yunnanensis* | 0±0 | 0.03±0.01 | 8.57 | 0.06 |
| **Class Betaproteobacteria** | 45.34±42.55 | 12.58±1.32 | 2.13 | 0.24 |
| Order Burkholderiales | 44.44±43.63 | 6.27±0.5 | 2.75 | 0.20 |
| Family Burkholderiaceae | 44.41±43.62 | 5.51±0.49 | 2.86 | 0.19 |
| *Burkholderia* | 44.28±43.43 | 5.51±0.51 | 2.87 | 0.19 |
| *Burkholderia cepacia* complex\|*Burkholderia lata* | 44.28±43.43 | 5.51±0.51 | 2.87 | 0.19 |
| *Caballeronia* | 0.13±0.18 | 0.01±0.01 | 1.51 | 0.31 |
| *Burkholderia megalochromosomata* | 0.13±0.18 | 0.01±0.01 | 1.51 | 0.31 |
| Family Comamonadaceae | 0±0 | 0.43±0.36 | 2.52 | 0.21 |
| *Ramlibacter* | 0±0 | 0.43±0.36 | 2.52 | 0.21 |
| *Ramlibacter solisilvae* | 0±0 | 0.43±0.36 | 2.52 | 0.21 |
| Family Oxalobacteraceae | 0.02±0.02 | 0.33±0.51 | 0.66 | 0.48 |
| *Massilia* | 0.02±0.02 | 0.33±0.51 | 0.66 | 0.48 |
| *Massilia consociata* | 0.02±0.02 | 0.33±0.51 | 0.66 | 0.48 |
| Order Neisseriales | 0.9±1.08 | 6.31±1.82 | 13.51 | 0.03* |
| Family Chromobacteriaceae | 0.9±1.08 | 6.31±1.82 | 13.51 | 0.03* |
| *Jeongeupia* | 0.9±1.08 | 6.31±1.82 | 13.51 | 0.03* |
| *Jeongeupia chitinilytica* | 0.9±1.08 | 6.31±1.82 | 13.51 | 0.03* |
| **Class Deltaproteobacteria** | 0.14±0.19 | 0.09±0.15 | 0.10 | 0.77 |
| Order Myxococcales | 0.14±0.19 | 0.09±0.15 | 0.10 | 0.77 |
| Family Cystobacterineae | 0.14±0.19 | 0.09±0.15 | 0.10 | 0.77 |
| Cystobacteraceae | 0.14±0.19 | 0.09±0.15 | 0.10 | 0.77 |
| *Cystobacter*\|*Cystobacter velatus* | 0.14±0.19 | 0.09±0.15 | 0.10 | 0.77 |
| Class Gammaproteobacteria | 0.23±0.17 | 0.34±0.24 | 0.33 | 0.61 |
| Order Oceanospirillales | 0.21±0.2 | 0.03±0 | 3.02 | 0.18 |
| Family Halomonadaceae | 0.21±0.2 | 0.03±0 | 3.02 | 0.18 |
| *Halomonas* | 0.21±0.2 | 0.03±0 | 3.02 | 0.18 |
| *Halomonas stevensii*\|*Halomonas stevensii* S18214 | 0.21±0.2 | 0.03±0 | 3.02 | 0.18 |
| Order Pseudomonadales | 0±0 | 0.31±0.24 | 3.14 | 0.17 |
| Family Moraxellaceae | 0±0 | 0.13±0.22 | 0.60 | 0.50 |
| *Moraxella* | 0±0 | 0.13±0.22 | 0.60 | 0.50 |
| *Moraxella osloensis* | 0±0 | 0.13±0.22 | 0.60 | 0.50 |
| Family Pseudomonadaceae | 0±0 | 0.18±0.23 | 1.22 | 0.35 |
| *Pseudomonas* | 0±0 | 0.18±0.23 | 1.22 | 0.35 |
| *Pseudomonas putida* group\|*Pseudomonas oryzihabitans* | 0±0 | 0.18±0.23 | 1.22 | 0.35 |
| Order Xanthomonadales | 0.02±0.03 | 0±0 | 1.80 | 0.27 |
| Family Xanthomonadaceae | 0.02±0.03 | 0±0 | 1.80 | 0.27 |
| *Stenotrophomonas* | 0.02±0.03 | 0±0 | 1.80 | 0.27 |
| *Stenotrophomonas maltophilia* group\|*Stenotrophomonas maltophilia*\|*Stenotrophomonas maltophilia* R551-3 | 0.02±0.03 | 0±0 | 1.80 | 0.27 |
| No blast hit | 23.64±17.73 | 10.49±7.12 | 1.50 | 0.31 |
