# Supplementary material for: Core members and differential abundance of chrysomelid microbiota in the life stages of Podontiaaffinis (Galerucinae) and adult Silanafarinosa (Cassidinae, Coleoptera)
Source: Biodivers Data J. 2022 Oct 7;10:e87459. doi: 10.3897/BDJ.10.e87459 (PMC9836631; doi:10.3897/BDJ.10.e87459)
Supplement: Supplementary material 6 — Comparisons of relative abundance of bacterial OTUs between the adult beetles of Podontiaaffinis and Salina farinosa [file bdj-10-e87459-s006.docx]

**Table S6**

Comparisons of relative abundance of bacterial OTUs between the adult beetles of *Podontia affinis* and *Salina farinosa*. *indicates significantly different.

|  | PA2 & PA3  Mean±SD | SF1-SF5  Mean±SD | ANOVA F-stat | p-value |
| --- | --- | --- | --- | --- |
| **Phylum Actinobacteria** | 0.51±0.58 | 0.01±0.01 | 5.26 | 0.07 |
| **Class Actinobacteria** | 0.51±0.58 | 0.01±0.01 | 5.26 | 0.07 |
| Order Micrococcales | 0.26±0.29 | 0.01±0.01 | 5.09 | 0.07 |
| Family Microbacteriaceae | 0.23±0.29 | 0.01±0.01 | 3.91 | 0.10 |
| *Curtobacterium* | 0.23±0.29 | 0±0 | 4.21 | 0.10 |
| *Curtobacterium oceanosedimentum* | 0.23±0.29 | 0±0 | 4.21 | 0.10 |
| *Leifsonia* | 0.12±0.17 | 0.01±0 | 3.32 | 0.13 |
| *Leifsonia shinshuensis* | 0.12±0.17 | 0.01±0 | 3.32 | 0.13 |
| Order Propionibacteriales | 0.03±0.04 | 0±0 | 7.00 | 0.05 |
| Family Propionibacteriaceae | 0.03±0.04 | 0±0 | 7.00 | 0.05 |
| *Propionibacterium* | 0.03±0.04 | 0±0 | 7.00 | 0.05 |
| *Propionibacterium acnes*\|*Propionibacterium acnes* KPA171202 | 0.03±0.04 | 0±0 | 7.00 | 0.05 |
| Order Pseudonocardiales | 0.19±0.23 | 0±0 | 4.49 | 0.09 |
| Family Pseudonocardiaceae | 0.19±0.23 | 0±0 | 4.49 | 0.09 |
| *Actinomycetospora* | 0.13±0.17 | 0±0 | 3.57 | 0.12 |
| *Actinomycetospora atypica* | 0.06±0.08 | 0±0 | 3.57 | 0.12 |
| *Actinomycetospora chiangmaiensis* | 0.07±0.09 | 0±0 | 3.57 | 0.12 |
| **Phylum Bacteroidetes** | 0.14±0.01 | 0±0 | 3003.57 | 0.00* |
| **Class Sphingobacteriia** | 0.11±0.01 | 0±0 | 432.14 | 0.00* |
| Order Sphingobacteriales | 0.11±0.01 | 0±0 | 432.14 | 0.00* |
| Family Sphingobacteriaceae | 0.11±0.01 | 0±0 | 432.14 | 0.00* |
| *Mucilaginibacter* | 0.06±0.06 | 0±0 | 8.04 | 0.04* |
| *Mucilaginibacter koreensis* | 0.05±0.07 | 0±0 | 3.57 | 0.12 |
| *Nubsella* | 0.05±0.07 | 0±0 | 3.57 | 0.12 |
| *Nubsella zeaxanthinifaciens* | 0.05±0.07 | 0±0 | 3.57 | 0.12 |
| **Phylum Cyanobacteria/Melainabacteria group** | 24.63±28.96 | 21.24±11.75 | 0.06 | 0.82 |
| **Class Cyanobacteria** | 24.57±29.05 | 21.24±11.75 | 0.06 | 0.82 |
| Order Stigonematales | 24.56±29.05 | 21.24±11.75 | 0.06 | 0.82 |
| Family Stigonemataceae | 24.56±29.05 | 21.24±11.75 | 0.06 | 0.82 |
| *Fischerella* | 13.43±15.77 | 11.63±6.41 | 0.06 | 0.82 |
| *Fischerella muscicola*\|*Fischerella muscicola* PCC 7414 | 0±0 | 0.02±0.01 | 4.47 | 0.09 |
| *Fischerella thermalis* | 13.43±15.77 | 11.61±6.4 | 0.06 | 0.82 |
| *Mastigocoleus* | 11.13±13.28 | 9.61±5.34 | 0.06 | 0.82 |
| *Mastigocoleus testarum* | 11.13±13.28 | 9.61±5.34 | 0.06 | 0.82 |
| **Class Cyanobacteria\|Oscillatoriophycideae** | 0.06±0.09 | 0±0 | 3.57 | 0.12 |
| Order Oscillatoriales | 0.06±0.09 | 0±0 | 3.57 | 0.12 |
| Family Pseudanabaenaceae | 0.06±0.09 | 0±0 | 3.57 | 0.12 |
| *Tapinothrix* | 0.04±0.05 | 0±0 | 3.57 | 0.12 |
| *Tapinothrix clintonii*\|*Tapinothrix clintonii* GSE-PSE06-07G | 0.04±0.05 | 0±0 | 3.57 | 0.12 |
| **Phylum Firmicutes** | 0.02±0.03 | 0.01±0.01 | 0.09 | 0.78 |
| **Class Clostridia** | 0.02±0.03 | 0.01±0.01 | 0.09 | 0.78 |
| Order Clostridiales | 0.02±0.03 | 0.01±0.01 | 0.09 | 0.78 |
| Family Heliobacteriaceae | 0.02±0.03 | 0.01±0.01 | 0.09 | 0.78 |
| *Heliorestis* | 0.02±0.03 | 0.01±0.01 | 0.09 | 0.78 |
| *Heliorestis acidaminivorans* | 0.02±0.03 | 0.01±0.01 | 0.09 | 0.78 |
| **Phylum Proteobacteria** | 50.99±46.08 | 65.23±12.72 | 0.52 | 0.50 |
| **Class Alphaproteobacteria** | 5.28±3.54 | 3.33±2.13 | 0.89 | 0.39 |
| Order Caulobacterales | 0.07±0.09 | 0±0 | 4.86 | 0.08 |
| Family Caulobacteraceae | 0.07±0.09 | 0±0 | 4.86 | 0.08 |
| *Phenylobacterium* | 0.07±0.09 | 0±0 | 4.86 | 0.08 |
| *Phenylobacterium koreense* | 0.07±0.09 | 0±0 | 4.86 | 0.08 |
| Order Rhizobiales | 3.88±4.84 | 0.36±0.47 | 3.64 | 0.11 |
| Family Bradyrhizobiaceae | 0.18±0.08 | 0.01±0 | 30.23 | 0.00* |
| *Bradyrhizobium* | 0.12±0.03 | 0±0 | 113.02 | 0.00* |
| *Bradyrhizobium ottawaense* | 0.12±0.03 | 0±0 | 113.02 | 0.00* |
| *Salinarimonas* | 0.06±0.05 | 0±0 | 7.33 | 0.04* |
| *Salinarimonas rosea* | 0.06±0.05 | 0±0 | 7.33 | 0.04* |
| Family Methylobacteriaceae | 3.66±4.81 | 0.34±0.46 | 3.28 | 0.13 |
| *Methylobacterium* | 3.66±4.81 | 0.34±0.46 | 3.28 | 0.13 |
| *Methylobacterium phyllostachyos* | 3.44±4.7 | 0.29±0.47 | 3.08 | 0.14 |
| *Methylobacterium tarhaniae* | 0.08±0.04 | 0.05±0.05 | 0.97 | 0.37 |
| Family Rhizobiaceae | 0.02±0.03 | 0.01±0.01 | 0.70 | 0.44 |
| *Agrobacterium* | 0.02±0.03 | 0.01±0.01 | 0.70 | 0.44 |
| *Agrobacterium larrymoorei* | 0.02±0.03 | 0.01±0.01 | 0.70 | 0.44 |
| Order Rhodospirillales | 1.09±1.29 | 2.9±1.85 | 1.51 | 0.27 |
| Family Rhodospirillaceae | 1.09±1.29 | 2.9±1.85 | 1.51 | 0.27 |
| *Limimonas* | 1.09±1.29 | 2.9±1.85 | 1.51 | 0.27 |
| *Limimonas halophila* | 1.09±1.29 | 2.9±1.85 | 1.51 | 0.27 |
| Order Sphingomonadales | 0.21±0.06 | 0.08±0.12 | 1.88 | 0.23 |
| Family Sphingomonadaceae | 0.21±0.06 | 0.08±0.12 | 1.88 | 0.23 |
| *Sphingomonas* | 0.21±0.06 | 0.08±0.12 | 1.88 | 0.23 |
| *Sphingomonas dokdonensis* | 0.03±0.01 | 0.01±0.01 | 9.29 | 0.03* |
| *Sphingomonas kyungheensis* | 0.07±0.03 | 0.02±0.02 | 6.27 | 0.05 |
| *Sphingomonas paucimobilis* | 0.02±0.02 | 0.06±0.09 | 0.34 | 0.58 |
| **Class Betaproteobacteria** | 45.34±42.55 | 4.86±3.02 | 6.33 | 0.05 |
| Order Burkholderiales | 44.44±43.63 | 2.44±2.26 | 6.55 | 0.05 |
| Family Burkholderiaceae | 44.41±43.62 | 2.24±1.89 | 6.63 | 0.05 |
| *Burkholderia* | 44.28±43.43 | 2.24±1.89 | 6.63 | 0.05 |
| *Burkholderia cepacia* complex\|*Burkholderia lata* | 44.28±43.43 | 2.24±1.89 | 6.63 | 0.05 |
| *Caballeronia* | 0.13±0.18 | 0±0 | 3.57 | 0.12 |
| *Burkholderia megalochromosomata* | 0.13±0.18 | 0±0 | 3.57 | 0.12 |
| Family Oxalobacteraceae | 0.02±0.02 | 0.2±0.4 | 0.38 | 0.57 |
| *Massilia* | 0.02±0.02 | 0.2±0.4 | 0.38 | 0.57 |
| *Massilia consociata* | 0.02±0.02 | 0.2±0.4 | 0.38 | 0.57 |
| Order Neisseriales | 0.9±1.08 | 2.43±1.53 | 1.59 | 0.26 |
| Family Chromobacteriaceae | 0.9±1.08 | 2.43±1.53 | 1.59 | 0.26 |
| *Jeongeupia* | 0.9±1.08 | 2.43±1.53 | 1.59 | 0.26 |
| *Jeongeupia chitinilytica* | 0.9±1.08 | 2.43±1.53 | 1.59 | 0.26 |
| **Class Deltaproteobacteria** | 0.14±0.19 | 0±0 | 3.57 | 0.12 |
| Order Myxococcales | 0.14±0.19 | 0±0 | 3.57 | 0.12 |
| Family Cystobacterineae | 0.14±0.19 | 0±0 | 3.57 | 0.12 |
| Cystobacteraceae | 0.14±0.19 | 0±0 | 3.57 | 0.12 |
| *Cystobacter*\|*Cystobacter velatus* | 0.14±0.19 | 0±0 | 3.57 | 0.12 |
| **Class Gammaproteobacteria** | 0.23±0.17 | 57.03±17.23 | 19.41 | 0.01* |
| Order Alteromonadales | 0±0 | 0.03±0.03 | 2.09 | 0.21 |
| Family Alteromonadaceae | 0±0 | 0.03±0.03 | 2.09 | 0.21 |
| *Aliagarivorans* | 0±0 | 0.03±0.03 | 2.09 | 0.21 |
| *Aliagarivorans marinus* | 0±0 | 0.03±0.03 | 2.09 | 0.21 |
| Order Enterobacteriales | 0.04±0.01 | 56.5±16.74 | 20.30 | 0.01* |
| Family Enterobacteriaceae | 0.04±0.01 | 56.5±16.74 | 20.30 | 0.01* |
| *Enterobacter* | 0±0 | 0.12±0.15 | 1.13 | 0.34 |
| *Enterobacter aerogenes*\|*Enterobacter aerogenes* KCTC 2190 | 0±0 | 0.12±0.15 | 1.13 | 0.34 |
| *Erwinia* | 0±0 | 0.21±0.31 | 0.79 | 0.41 |
| *Erwinia toletana* | 0±0 | 0.21±0.31 | 0.79 | 0.41 |
| *Hafnia* | 0±0 | 0.21±0.18 | 2.53 | 0.17 |
| *Hafnia alvei* | 0±0 | 0.16±0.16 | 1.99 | 0.22 |
| *Hafnia paralvei* | 0±0 | 0.04±0.06 | 1.04 | 0.35 |
| *Kluyvera* | 0±0 | 7.96±10.79 | 0.97 | 0.37 |
| *Kluyvera cryocrescens* | 0±0 | 7.96±10.79 | 0.97 | 0.37 |
| *Pantoea* | 0.03±0.02 | 22.41±22.09 | 1.83 | 0.23 |
| *Pantoea brenneri* | 0±0 | 0.03±0.04 | 1.02 | 0.36 |
| *Pantoea dispersa* | 0±0 | 0.02±0.02 | 0.81 | 0.41 |
| *Pantoea eucrina* | 0.01±0.01 | 0.28±0.42 | 0.73 | 0.43 |
| *Pantoea septica* | 0.02±0.01 | 22.09±21.91 | 1.81 | 0.24 |
| *Pseudocitrobacter* | 0.01±0.01 | 0.09±0.09 | 1.55 | 0.27 |
| *Pseudocitrobacter anthropi* | 0.01±0.01 | 0.09±0.09 | 1.55 | 0.27 |
| *Raoultella* | 0±0 | 0.06±0.09 | 0.79 | 0.42 |
| *Raoultella planticola*\|*Raoultella planticola* ATCC 33531 | 0±0 | 0.06±0.09 | 0.79 | 0.42 |
| *Rosenbergiella* | 0±0 | 0.2±0.18 | 2.31 | 0.19 |
| *Rosenbergiella collisarenosi* | 0±0 | 0.02±0.02 | 1.25 | 0.31 |
| *Rosenbergiella epipactidis* | 0±0 | 0.19±0.16 | 2.30 | 0.19 |
| unclassified Enterobacteriaceae | 0±0 | 25.25±17.68 | 3.64 | 0.11 |
| Secondary endosymbiont of Heteropsylla cubana | 0±0 | 25.25±17.68 | 3.64 | 0.11 |
| Order Oceanospirillales | 0.21±0.2 | 0.01±0 | 7.45 | 0.04* |
| Family Halomonadaceae | 0.21±0.2 | 0.01±0 | 7.45 | 0.04* |
| *Halomonas* | 0.21±0.2 | 0.01±0 | 7.45 | 0.04* |
| *Halomonas stevensii*\|*Halomonas stevensii* S18214 | 0.21±0.2 | 0.01±0 | 7.45 | 0.04* |
| Order Pseudomonadales | 0±0 | 0.45±0.59 | 1.07 | 0.35 |
| Family Pseudomonadaceae | 0±0 | 0.45±0.59 | 1.07 | 0.35 |
| *Pseudomonas* | 0±0 | 0.45±0.59 | 1.07 | 0.35 |
| *Pseudomonas putida* group\|*Pseudomonas oryzihabitans* | 0±0 | 0.45±0.59 | 1.07 | 0.35 |
| Order Vibrionales | 0±0 | 0.04±0.04 | 1.20 | 0.32 |
| Family Vibrionaceae | 0±0 | 0.04±0.04 | 1.20 | 0.32 |
| *Photobacterium* | 0±0 | 0.04±0.04 | 1.20 | 0.32 |
| *Photobacterium leiognathi*\|*Photobacterium leiognathi* subsp. *mandapamensis* | 0±0 | 0.04±0.04 | 1.20 | 0.32 |
| No blast hit | 23.64±17.73 | 13.5±7.84 | 1.31 | 0.30 |
